# p85S6K sustains synaptic GluA1 to ameliorate cognitive deficits in Alzheimer’s disease

**DOI:** 10.1186/s40035-022-00334-w

**Published:** 2023-01-09

**Authors:** Jia-Bing Li, Xiao-Yu Hu, Mu-Wen Chen, Cai-Hong Xiong, Na Zhao, Yan-Hui Ge, Hao Wang, Xiao-Ling Gao, Nan-Jie Xu, Lan-Xue Zhao, Zhi-Hua Yu, Hong-Zhuan Chen, Yu Qiu

**Affiliations:** 1 Department of Pharmacology and Chemical Biology, Shanghai Jiao Tong University School of Medicine, Shanghai, 200025 China; 2 Institute of Interdisciplinary Integrative Biomedical Research, Shuguang Hospital, Shanghai University of Traditional Chinese Medicine, Shanghai, 201210 China; 3 Collaborative Innovation Center for Brain Science, Department of Anatomy and Physiology, Shanghai Jiao Tong University School of Medicine, Shanghai, 200025 China

**Keywords:** Alzheimer’s disease, Cognition, GluA1 subunit of AMPA receptors, Ribosomal S6 protein kinase 1, 85 kDa isoform

## Abstract

**Background:**

Ribosomal protein S6 kinase 1 (S6K1) is a serine–threonine kinase that has two main isoforms: p70S6K (70-kDa isoform) and p85S6K (85-kDa isoform). p70S6K, with its upstream mammalian target of rapamycin (mTOR), has been shown to be involved in learning and memory and participate in the pathophysiology of Alzheimer’s disease (AD). However, the function of p85S6K has long been neglected due to its high similarity to p70S6k. The role of p85S6K in learning and memory is still largely unknown.

**Methods:**

We fractionated the postsynaptic densities to illustrate the differential distribution of p85S6K and p70S6K. Coimmunoprecipitation was performed to unveil interactions between p85S6K and the GluA1 subunit of AMPA receptor. The roles of p85S6K in synaptic targeting of GluA1 and learning and memory were evaluated by specific knockdown or overexpression of p85S6K followed by a broad range of methodologies including immunofluorescence, Western blot, in situ proximity ligation assay, morphological staining and behavioral examination. Further, the expression level of p85S6K was measured in brains from AD patients and AD model mice.

**Results:**

p85S6K, but not p70S6K, was enriched in the postsynaptic densities. Moreover, knockdown of p85S6K resulted in defective spatial and recognition memory. In addition, p85S6K could interact with the GluA1 subunit of AMPA receptor through synapse-associated protein 97 and A-kinase anchoring protein 79/150. Mechanistic studies demonstrated that p85S6K could directly phosphorylate GluA1 at Ser845 and increase the amount of GluA1 in synapses, thus sustaining synaptic function and spine densities. Moreover, p85S6K was found to be specifically decreased in the synaptosomal compartment in the brains of AD patients and AD mice. Overexpression of p85S6K ameliorated the synaptic deficits and cognitive impairment in transgenic AD model mice.

**Conclusions:**

These results strongly imply a significant role for p85S6K in maintaining synaptic and cognitive function by interacting with GluA1. The findings provide an insight into the rational targeting of p85S6K as a therapeutic potential for AD.

**Supplementary Information:**

The online version contains supplementary material available at 10.1186/s40035-022-00334-w.

## Background

Ribosomal protein S6 kinase 1 (S6K1) belongs to the AGC kinase family, which is a group of kinases related to protein kinase A (PKA), cGMP-dependent protein kinase/protein kinase G and protein kinase C, whose members are serine–threonine kinases and share some structural features. S6K1 has two main isoforms transcribed from a single gene: p70S6K (70-kDa isoform) and p85S6K (85-kDa isoform) [1]. Both isoforms are considered to be downstream effectors of mammalian target of rapamycin (mTOR) [2, 3]. Compared to p70S6K, p85S6K contains additional 23 amino acids in the N-terminus with a putative nuclear localization signal (NLS). Although early work suggested nuclear localization of p85S6K [4], recent studies have identified p85S6K to be primarily cytoplasmic [5, 6], whereas p70S6K resides in both cytoplasm and the nucleus [6, 7]. Although both isoforms can phosphorylate the S6 protein and are supposed to mediate protein translation, only p70S6K is shown to be able to translocate to the nucleus after activation in the mid G1 phase [6]. Thus, p70S6K and p85S6k may possess different functions. However, the function of p85S6K has long been neglected due to its high similarity to p70S6k.

p70S6K, together with its upstream mTOR, has received attention as a key modulator in memory and Alzheimer’s disease (AD) [8–12], the most prevalent neurodegenerative disease characterized by progressive cognitive dysfunction. p70S6K as a ubiquitously expressed kinase involved in protein translation and cell proliferation, is required for synaptic function and memory formation [13]. Mice undertaking learning tasks display activated p70S6K in task-related brain regions [14, 15], consistent with its role in protein synthesis in long-term synaptic plasticity [16, 17]. On the other hand, hyperactive p70S6K and mTOR have been shown in brain regions affected by AD in mouse models and patients [18, 19]. Furthermore, cognitive performance negatively correlates with p70S6K signaling in AD mice [12]. In these studies, the role of p85S6K was neglected. Besides, immunofluorescence using antibodies recognizing both isoforms cannot experimentally differentiate between p70S6K and p85S6K [20, 21]. Thus, whether and how p85S6K is involved in learning and memory is still elusive. In this study, we aimed to investigate the role of p85S6K in learning and memory, and possibly its role in AD, thereby advancing our understanding of S6K1 in maintaining synaptic and cognitive function.

## Methods

### Animals and ethics statement

Wild-type (WT) C57BL/6J male mice and 24-h newborn Sprague–Dawley (SD) rats were purchased from SLAC Laboratory Animal Co. Ltd. (Shanghai, China). APP/PS1 (APPswe and PSEN1dE9 mutation) male mice with C57BL/6J background were from the Nanjing Biomedical Research Institute of Nanjing University (Nanjing, China).  5×FAD male mice were from the Jackson Laboratory (Bar Harbor, ME). Heterozygous GluA1-mutated transgenic mice in which Ser845 of GluA1 is mutated to Ala (S845A) (gifts from Dr. Hey-Kyoung Lee, the Johns Hopkins School of Medicine, USA) were breed to obtain homozygous ones. All animal procedures were approved by the Institutional Animal Care and Use Committee of Shanghai Jiao Tong University School of Medicine.

### Human brain samples

Postmortem temporal cortex samples from AD patients and non-demented controls were obtained from The Netherlands Brain Bank, Netherlands Institute for Neuroscience, Amsterdam (open access: www.brainbank.nl). Written informed consents for a brain autopsy and the use of the material and clinical information for research purposes had been obtained by The Netherlands Brain Bank. Detailed information of the brain donors is described in Additional file 1: Table S1.

### Plasmids and viruses

Plasmids expressing Myc-GluA1 or Myc-GluA2 were kindly provided by Dr. Alex L. Kolodkin from The Johns Hopkins School of Medicine. AKAP79-GFP plasmids were kindly provided by Dr. Mark L. Dell'Acqua from University of Colorado School of Medicine. Plasmids expressing Flag-p85S6K, Flag-p70S6K or GST-GluA1 C-terminus (residues 810–889) in PcDNA3.1 were purchased from SunBio Technology (Shanghai, China) and verified by DNA sequencing. Lentiviruses expressing p70S6K, p85S6K or Flag-p85S6KT421A were from OBiO Technology (Shanghai, China). Adeno-associated viruses (AAVs, serotype 2/9) expressing p70S6K, p85S6K, or shRNA for S6K1 (target sequence: CCTTTCAGACCGGAGGAAA for mice), and lentiviruses expressing S6K1 shRNA (target sequence: GCACCTGCGTATGAATCTA for rats) and corresponding controls were from SunBio Technology. The control sequence for shRNA was TTCTCCGAACGTGTCACGT. All AAVs were with the synapsin-1 promoter.

### Cell line and transfection

HEK293 cells (ATCC, Manassas, VA, Cat# CRL-1573™) were maintained with DMEM containing 50 U/ml penicillin and streptomycin, and 10% fetal bovine serum, in a humidified incubator at 37 °C with 5% CO_2_. Cells were transfected with plasmids using Lipofectamine 3000 (Thermo Fisher Scientific, Waltham, MA) according to the manufacture’s instruction, and processed 48 h after transfection.

### Primary hippocampal neuron culture and infection

Primary hippocampal neurons were obtained from the hippocampus of newborn SD rats at 24 h after birth as previously described [22]. Neurons were plated onto a 35-mm glass-bottom petri dish at a density of 1 × 10^6^ cells/dish or onto a 6-well plate coated with poly-*L*-lysine (100 µg/ml, Millipore Sigma, Burlington, MA) at a density of 1 × 10^6^ cells/well. The neurons were used for experiments between culture days 18 and 24. The neurons were infected on day 9 with lentiviruses. For specific knockdown of p85S6K, lentiviruses expressing S6K1 shRNA and p70S6K were administered simultaneously in a 4:1 viral particle ratio. The control neurons were infected with corresponding control AAVs.

### Hippocampal injection

The mice were anesthetized by inhalation of 2% isoflurane. To ensure better coverage of the whole hippocampus, each hippocampus was given two injections at positions stereotaxically defined as (1) 1.95 mm posterior to the bregma, 1.0 mm lateral to the midline and 2.2 mm ventral to the skull surface, and (2) 2.9 mm posterior to the bregma, 3.0 mm lateral to the midline, and 3.2 mm ventral to the skull surface. One microliter of AAV particles was slowly injected over 10 min and then left for 10 min to facilitate diffusion. The needle was slowly raised over a 2-min period. For specific knockdown of p85S6K, AVVs expressing S6K1 shRNA and p70S6K were co-administered in a 2.5:1 viral particle ratio. The control mice were injected with corresponding control AAVs. Subsequent experiments were carried out 2 months after viral injection.

### Postsynaptic density (PSD) fractionation

The pool of PSDs was prepared as previously described [23]. Hippocampi from 10 mice were homogenized in an ice-cold homogenization buffer containing 0.32 M sucrose, 4 mM HEPES, 2 mM EDTA, 50 mM NaF, 1 mM sodium orthovanadate, 0.1 mg/ml benzamidine, and a protease inhibitor cocktail (APExBIO Technology, Shanghai, China), pH 7.4. The homogenates were centrifuged at 1000 g for 10 min to obtain supernatant S1. The S1 was spun at 10,000 g for 15 min to yield the crude synaptosome pellet (P2) and the supernatant S2. The P2 pellet was washed, lysed, and centrifuged at 25,000 g for 20 min to yield a lysed synaptosome membrane fraction pellet. The resulting pellet was resuspended in HEPES-buffered sucrose (0.32 M sucrose, 4 mM HEPES, pH 7.4) and further fractionated by a discontinuous sucrose gradient (0.8, 1.0, and 1.2 M sucrose) at 150,000 g for 2 h in a swinging bucket rotor (SW32). The synaptosomes in the layer between 1.0 and 1.2 M sucrose were recovered and further lysed and centrifuged at 32,000 g for 20 min to obtain the PSD-1 pellet. Non-synaptosome fraction in the layer between 0.8 and 1.0 M sucrose was saved. The PSD-1 pellet was further lysed in 0.5% Triton X-100 and centrifuged at 200,000 g for 15 min to obtain the PSD-2 pellet. The pellets were resuspended in a buffer containing 50 mM HEPES, 2 mM EDTA, and protease inhibitor cocktail, pH 7.4. All the fractions were subsequently subjected to SDS-PAGE and western blotting analysis.

### Synaptosome trypsin cleavage assay

Trypsin treatment of synaptosomes was performed as previously described [24]. Briefly, the synaptosomal compartment (P2 pellet of the above fractionation) was resuspended in ice-cold sucrose buffer [320 mM sucrose and 5 mM HEPES (pH 8)]. Then a trypsin stock solution (0.1 mg/ml) was added to yield a final protein-protease ratio of 100:1. Synaptosomes were incubated for 30 min at 30 °C with gentle agitation. The mixture was centrifuged for 3 min at 8700 g. The resulting pellet was resuspended in 1 × SDS sample buffer and then subjected to SDS-PAGE and western blotting analysis.

### Co-immunoprecipitation

Cultured cells were lysed in Pierce™ IP lysis buffer (Thermo Fisher Scientific) with a protease inhibitor cocktail (APExBIO Technology). Hippocampi from mice were dissected, homogenized, and solubilized at 4 °C for 1 h in IP lysis buffer with protease inhibitors. The PSD-1 pellet from PSD fractionation was also lysed in Pierce™ IP lysis buffer. Five hundred micrograms of protein of the lysates were added with 1 μg antibody (mouse anti-Flag (M2) [#F1804, Millipore Sigma], mouse anti-c-Myc (9E10) [#M4439, Millipore Sigma], mouse anti-GluA1 [#MAB2263, Millipore Sigma], and rabbit anti-p70S6K [#9202, Cell Signaling Technology, Danvers, MA]) and incubated at 4 °C overnight. Next, the samples were incubated with protein A/G agarose resin (Santa Cruz Biotechnology, Dallas, TX) with rotation for 2 h. Proteins were eluted from the resin with 2 × SDS sample buffer and then subjected to SDS-PAGE and western blotting analysis. For multiple blotting, eluates were split equally and subjected to SDS-PAGE separately.

### Immunofluorescence staining

The cultured neurons were incubated with rabbit anti-GluA1 N-terminus (1:100; #PC246, Millipore Sigma) and then fixed in 2% paraformaldehyde. The cells were blocked and permeabilized with PBS containing 10% normal goat serum and 1% Triton X-100 for 2 h at room temperature. Then, the slices were stained with mouse anti-PSD95 (1:1000; #sc-32291, Santa Cruz Biotechnology) overnight at 4 °C and subsequently stained with Alexa 488-conjugated goat anti-rabbit (1:1000, Jackson ImmunoResearch, West Grove, PA) and Alexa 633-conjugated goat anti-mouse antibodies (1:2000, Jackson ImmunoResearch). Images were captured using a Leica TCS SP8 laser confocal microscope (Leica Microsystems, Buffalo Grove, IL) and colocalization of GluA1 and PSD95 was analyzed using ImageJ software (National Institutes of Health, Bethesda, MA). For staining of endogenous S6K1 or exogenous p70S6K and p85S6K in neurons and HEK293 cells, the cells were fixed first. After permeabilization and blocking, the cells were then incubated with anti-p70S6K or anti-Flag antibody.

### Surface biotinylation

Surface proteins of primary hippocampal neurons were biotinylated with 1 mg/ml sulfo-NHS-LC-biotin (Thermo Fisher Scientific) according to the manufacturer’s instructions. The unbound biotin was washed away by PBS/Ca^2+^/Mg^2+^ containing 0.1% BSA at 4 °C. Cells were then solubilized in lysis buffer (50 mM Tris, pH 7.4, 150 mM NaCl, 1 mM EDTA, 1% Triton X-100, 0.5% sodium deoxycholate, 30 mM NaF, 1 mM sodium orthovanadate and protease inhibitor cocktail [APExBIO Technology]). Thirty microliters of each lysate were used to determine the total GluA1, and the remaining lysate was incubated with streptavidin beads (Thermo Fisher Scientific) to detect surface GluA1 using rabbit anti-GluA1 antibody (1:1000; Abcam, Cambridge, United Kingdom).

### Western blotting

Cells were solubilized as above. Mouse cortex and hippocampal tissues stored in liquid nitrogen were thawed on ice and lysed in RIPA buffer containing 10 mM Tris–Cl (pH 8), 1 mM EDTA, 0.5 mM EGTA, 1% Triton X-100, 0.1% sodium deoxycholate, 0.1% SDS, 140 mM NaCl, and 1 mM PMSF and a protease inhibitor and phosphatase inhibitor cocktail (APExBIO Technology). The extracts were centrifuged at 12,000 g for 20 min at 4 °C. Equal amounts of proteins were subjected to SDS-PAGE and transferred to a polyvinylidene fluoride membrane (Millipore Sigma). The following primary antibodies were used: rabbit anti-p70S6K phosphorylated at Thr389 (1:1000; #9205, Cell Signaling Technology, or #MAB8963, R&D systems, Minneapolis, MN), rabbit anti-p70S6K (1:1000; #9202, Cell signaling Technology), rabbit anti-S6 (1:1000, #AF6354, Affinity Biosciences, Changzhou, China), rabbit anti-S6 phosphorylated at Ser235 (1:1000, #AF3354, Affinity Biosciences), mouse anti-SAP97 (1:500; #sc-9961, Santa Cruz Biotechnology), mouse anti-AKAP79 (1:500; #sc-17772, Santa Cruz Biotechnology), mouse anti-AKAP150 (1:500; #sc-377055, Santa Cruz Biotechnology), rabbit anti-GluA1 phosphorylated at Ser845 (1:1000; #ab76321, Abcam), rabbit anti-GluA1 (1:1000; #ab109450, Abcam), rabbit anti-GluA2 (1:1000; #ab133477, Abcam), rabbit anti-GluA3 (1:1000; #4676, Cell Signaling Technology), mouse anti-synaptophysin (1:1000; #sc-17750, Santa Cruz Biotechnology), mouse anti-PSD95 (1:1000; #sc-32291, Santa Cruz Biotechnology), rabbit anti-Flag (1:1000; #8146, Cell Signaling Technology), rabbit anti-Myc (1:1000; #2278, Cell Signaling Technology), rabbit anti-PKA catalytic subunit α (PKA C-α, 1:1000; #5842, Cell Signaling Technology) and rabbit anti-GAPDH (1:1000; #G9545, Millipore Sigma). Secondary antibodies were IRDye goat anti-rabbit or -mouse IgG (H + L) (Li-Cor Biosciences, Lincoln, NE). The bands were visualized and analyzed by Li-Cor Odyssey Fc Image Studio (Li-Cor Biosciences). Immunofluorescent intensity values were normalized to the corresponding total protein or GAPDH. For proteins with similar molecular weights to be blotted with antibodies of the same species of origin, the lysates were split equally and subjected to SDS-PAGE separately.

### In situ proximity ligation assay (PLA)

The brain slices were prepared according to previous descriptions with modifications [25]. The anesthetized mice were perfused with 250 ml of cold saline and 250 ml of 4% paraformaldehyde. The entire brain was soaked in 4% paraformaldehyde at 4 °C, then dehydrated with 10%, 20% and 30% sucrose solution until it sank to the bottom of the tube. The brain was then completely embedded with Tissue-Tek® O.C.T. Compound (Sakura Finetek, Torrance, CA), frozen at − 20 °C, and cut into 30-μm-thick sections. The brain slices were permeabilized with 0.1% Triton X-100 for 10 min at room temperature. Then PLA was performed using Duolink in situ PLA Kit Mouse/Rabbit (Red) (Millipore Sigma) according to the manufacturer's instructions. Mouse anti-GluA1 (#MAB2263, Millipore Sigma), mouse anti-GluA2 (#sc-517265, Santa Cruz Biotechnology) and rabbit anti-p70S6K (detecting both p85S6K and p70S6K, #9202, Cell Signaling Technology) antibodies were used. Images were taken using a Leica TCS SP8 laser confocal microscope.

### Synaptic PLA (SYNPLA)

SYNPLA was used to detect synaptic insertion of GluA1-containing AMPA receptors as previously described with mild modifications [26, 27]. Dore et al. used presynaptic binding protein neurexin 1b and GluA1 to detect the synaptic insertion of GluA1 [26]. Heaney et al. used a similar PLA assay with presynaptic protein synapsin-1 and postsynaptic PSD95 to detect synaptic changes [27]. Here, we used the presynaptic protein synaptophysin to detect synaptic insertion of GluA1. Brain slices were prepared according to previous descriptions [28]. Briefly, the brain was collected and immersed in cold oxygenated (equilibrated with 95% O_2_ and 5% CO_2_) artificial cerebrospinal fluid (ACSF, 118 mM NaCl, 2.5 mM KCl, 26 mM NaHCO_3_, 1 mM NaH_2_PO_4_, 1 mM MgCl_2_, 2 mM CaCl_2_, and 20 mM glucose). Transverse hippocampal slices of 300-μm thickness were cut and allowed to recover for at least 2 h in 30 °C ACSF. Then chemical long-term potentiation (LTP) was induced in the hippocampal slices using MgCl_2_-free ACSF containing100 nM rolipram, 50 µM forskolin and 100 µM picrotoxin for 16 min, and then immediately fixed in 4% paraformaldehyde. Then the PLA was performed with Duolink in situ PLA Kit using rabbit anti-GluA1 (#PC246, Millipore Sigma) and mouse anti-synaptophysin (#sc-17750, Santa Cruz Biotechnology) antibodies following the manufacturer's instructions. Images were captured using the same settings by a Leica TCS SP8 laser confocal microscope with a 63 × oil objective. Three z stacks (1 μm) of 1024 × 1024 pixels from ∼2 to 3 μm below the slice surface were acquired in the CA1 region for each slice. PLA puncta in all images were identified using the same settings by ImageJ. The dendrites at > 15 µm away from the soma were selected for analysis of PLA puncta.

### In vitro phosphorylation assay

The GST-GluA1 C-terminus expressed in HEK293 cells was pulled down by glutathione agarose (Santa Cruz Biotechnology), and mixed with Flag-p85S6K immunoprecipitated from HEK293 cell extracts in phosphorylation assay buffer (25 mM HEPES, pH 7.5, 2.5 mM EDTA, 7.5 mM MgCl_2_ and a protease inhibitor cocktail) supplemented with or without 1 mM ATP. After incubation at 30 °C for 30 min, the reaction was terminated by 2 × SDS sample buffer and then subjected to SDS-PAGE and western blotting analysis.

### Golgi staining and spine analysis

Golgi staining was performed to detect the dendritic spines of neurons using the FD Rapid Golgi Stain™ Kit (FD Neuro Technologies, Ellicott City, MD) following the manufacturer's instructions. Brain tissue sections of 100-μm thickness were cut, and dehydrated in sequential rinses of 50%, 75%, 95% and 100% ethanol and cleared in xylene. The staining was viewed under a Leica DFC320 microscope. ImageJ software was used to analyze the number of spines and the total dendritic length. Distal dendrites at > 50 µm away from the soma were selected for measurement of spine density.

### Behavioral tests

Male WT mice of 2 to 3 months old were used for behavioral assessment involving p85S6K knockdown. Male APP/PS1, S845A and WT male mice of 7 months old were used for behavioral assessment involving p85S6K overexpression. Behavioral tests were performed 2 months after AAV injection.

### Open field test

Open field test was performed to assess the motor activity and anxiety behavior. The mouse was placed in the center of an open field apparatus (30 cm × 30 cm × 30 cm) and allowed to freely explore it for 10 min. The movement of the mouse was recorded and analyzed by a video tracking system (Mobile Datum, Shanghai, China).

### Morris water maze (MWM)

The MWM procedure was performed as previously described with mild modifications [23, 29]. The mice were trained in a circular pool (150-cm diameter) filled with opaque water (30 cm deep, 19–22 °C). A 6-cm white platform was placed 1 cm below the surface in the middle of a specific quadrant. During 6-day training period, the mice were required to locate the hidden submerged platform for four trials. The mice that failed to find the platform were guided to the platform and stayed there for 30 s. On the last day for probe test, mice were required to navigate in the pool for 30 s without the platform. A video-tracking system (Mobile Datum) was used to record and analyze the swimming path and escape latencies.

### Novel object recognition (NOR)

The NOR procedure was used to investigate non-spatial memory performed as previously described with mild modifications [30]. The mice were habituated in an open field apparatus (30 cm × 30 cm × 30 cm) for 5 min on the first day. On the second day, the mice were placed in the apparatus for 10 min with two identical objects (training trial). In the testing trial 24 h later, the mice were placed again for 10 min in the apparatus with one original familiar object and one new object of different shape, size and material. The discrimination index was calculated as the time spent exploring the novel object minus the time spent exploring the familiar object divided by total exploration time.

### Statistics

Data are presented as the means ± standard error of the mean (SEM). The data were analyzed by GraphPad Prism 8.0. The comparison between two groups was conducted using unpaired two-tail Student’s *t*-test (*t* value, degree of freedom [df] and *P* value are provided). Comparisons among 3 or more groups were performed using one-way, two-way or three-way ANOVA followed by *post-hoc* analysis where appropriate (*F* and *P* values are provided). Data that are not normally distributed were analyzed with the non-parametric two-tailed Mann–Whitney test between two groups (Mann–Whitney *U* and *P* values are provided). Pearson correlation was used to assess the correlations of p85S6K level with Braak stage and colocalization of GluA1 and PSD95. *P* < 0.05 was considered statistically significant.

## Results

### p85S6K is enriched in PSDs and plays an important role in cognition

**Fig. 1:**
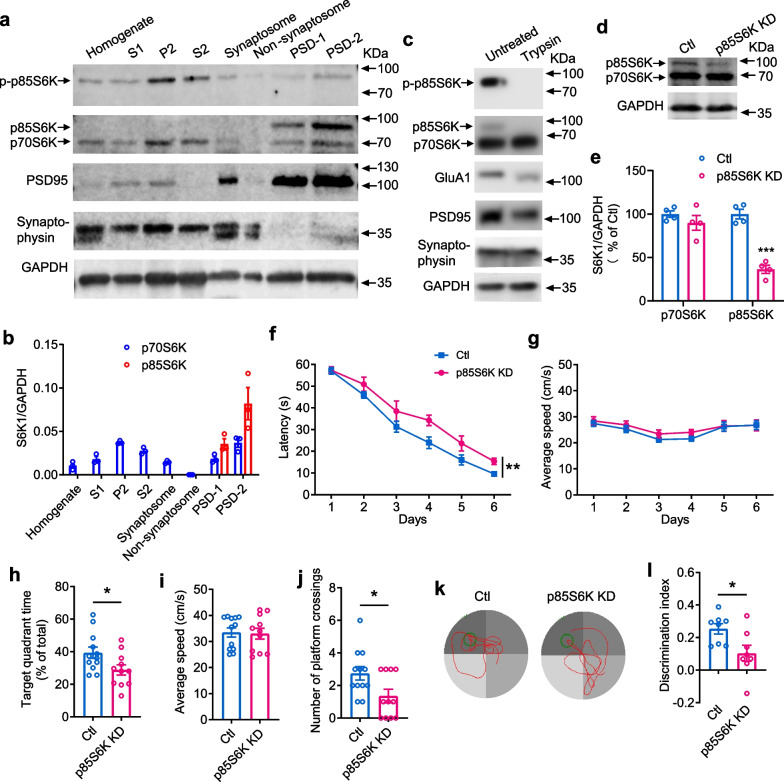
p85S6K is fractionated into PSDs and is involved in spatial and recognition memory. **a**, **b** Fractionation of PSDs from mouse hippocampus showed enrichment of p85S6K but not p70S6K in PSDs. The indicated subcellular fractions (8 µg protein for Syn and non-Syn fractions and 10 µg protein for other fractions) were analyzed by immunoblotting with anti-phosphorylated p70S6K, anti-p70S6K, anti-PSD95, anti-synaptophysin, and anti-GAPDH antibodies. *n* = 3 independent experiments. p-p85S6K: phosphorylated p85S6K. **c** Synaptosome trypsin digestion showed postsynaptic localization of p85S6K. Representative blots or images of 3 independent experiments are shown. **d**, **e** The expression of p85S6K and p70S6K after knockdown of p85S6K (p85S6K KD) in hippocampus of WT mice. Control mice were injected with AAVs expressing control shRNA. *n* = 4 mice per group. *t* = 8.835, df = 6, *P* = 0.0001. **f–k** Morris water maze was performed to examine effects of downregulation of p85S6K on spatial learning and memory. *n* = 12 mice for control and *n* = 11 mice for p85S6K KD. **f** The latency of mice to locate the hidden platform in the training period. *F*_(1, 21)_ = 9.742, *P* = 0.0052 for p85S6K expression manipulation. **g** The average speed of mice in the training period. **h** The time in the target quadrant in the probe test. *t* = 2.257, df = 21, *P* = 0.0348. **i** The average speed of mice in the probe test. **j** The number of platform crossings in the probe test. *t* = 2.383, df = 21, *P* = 0.0267. **k** Representative swimming trajectories in the probe test from different groups of mice. The green circle represents the hidden platform. **l** Discrimination index in the NOR test. *n* = 8 mice per group. *t* = 2.550, df = 14, *P* = 0.0231. Data are presented as mean ± SEM. Unpaired *t* test, two-tailed (**e, h–j, l**) and repeated measures two-way ANOVA followed by Tukey's test (**f**, **g**). Ctl, control. *P < 0.05, ***P < 0.001

First, we examined whether the distribution of p85S6K is different from that of p70S6K. Exogenous expression of p85S6K and p70S6K demonstrated that they were expressed similarly in cytosol and nucleus in HEK293 cells (Additional file 1: Fig. S1a). Immunostaining of S6K1 with the antibody recognizing both isoforms showed that the endogenous S6K1 was expressed throughout the cell body, nucleus and processes, including the spines on dendrites in neurons (Additional file 1: Fig. S1b). Knockdown of S6K1 weakened the immunostaining of S6K1 obviously (Additional file 1: Fig. S1c), confirming the staining of S6K1. Both p70S6K and p85S6K are downstream effectors of mTOR but they may reside in different compartments. To further define the distribution of p85S6K, we fractionated the synaptosomes and PSDs from hippocampi of mice. PSD95, the marker of PSDs, was shown to segregate fully to synaptosomal PSD-1 and PSD-2 fractions (Fig. 1a and Additional file 1: Fig. S2a), whereas synaptophysin, the marker of presynaptic compartments, segregated to the fractions other than PSDs (Fig. 1b and Additional file 1: Fig. S2b). p85S6K was substantially co-fractionated with PSD95 while not obviously detected in other fractions in the immunoblots with 8–10 µg protein. In contrast, p70S6K was much less detected in PSDs (Fig. 1a, b), because the antibody immunoblotted p70S6K and p85S6K similarly (Additional file 1: Fig. S2c). Moreover, p85S6K was more partitioned into the PSD-2 fraction (Fig. 1a, b), which comprised the purified insoluble PSDs [31]. The antibody for phosphorylated S6K1, which detected the phosphorylation state of both p70S6K and p85S6K similarly (Additional file 1: Fig. S2d), mainly detected the phosphorylated p85S6K in the homogenates and fractions (Fig. 1a), showing that the phosphorylation level of p85S6K is higher than that of p70S6K. These data demonstrate that p85S6K is abundantly present in PSDs, implicating its function in cognition. Moreover, p85S6K was hardly detected in P2 and S2 whereas the band intensity for phosphorylated p85S6K was much higher than that in PSDs (Fig. 1a), demonstrating higher phosphorylation levels of p85S6K in P2 and S2 fractions than in PSDs. This suggests that p85S6K needs a high phosphorylation level to functionally compete with p70S6K in synaptosomes and cytosol, while a lower phosphorylation level of p85S6K is allowable in PSDs due to its higher amount than p70S6K. Furthermore, the postsynaptic localization of p85S6K was further confirmed by the synaptosome trypsin cleavage assay. As expected, the presynaptic protein, synaptophysin, was protected from proteolysis (Fig. 1c), whereas the postsynaptic proteins PSD95 and GluA1 were sensitive to digestion with trypsin (Fig. 1c). p85S6K, in both total and phosphorylated forms, was also sensitive to tryptic digestion, while p70S6K was resistant to proteolysis (Fig. 1c). These data further demonstrate that p85S6K is mainly localized in PSDs.

Next, we knocked down the S6K1 gene and expressed p70S6K in the hippocampus to selectively reduce the expression of p85S6K (Fig. 1d, e; Additional file 1: Fig. S3a for co-expression of S6K1 shRNA and p70S6K, and Additional file 1: Fig. S3b for the knockdown efficiency in PSDs). Downregulation of p85S6K did not alter the locomotor activity and anxiety-like behavior as manifested in the open field test (Additional file 1: Fig. S3c-e). Then we tested the hippocampus-dependent spatial learning and memory by MWM and non-spatial memory by NOR [32]. In the MWM test, knockdown of p85S6K significantly slowed down the speed of mice to learn the location of the hidden platform during spatial learning, and reduced the time spent in the target quadrant and number of platform crossings in the probe test (Fig. 1f, h, j, k). The average swimming speed in the test did not differ among animals (Fig. 1g, i), indicating no effect of p85S6K knockdown on the locomotor function. In the NOR test, p85S6K knockdown resulted in decreased discrimination index at 24 h after training (Fig. 1l), indicating impairment of the recognition memory. These results indicate that p85S6K contributes to the maintenance of cognition.

### p85S6K interacts with GluA1

**Fig. 2:**
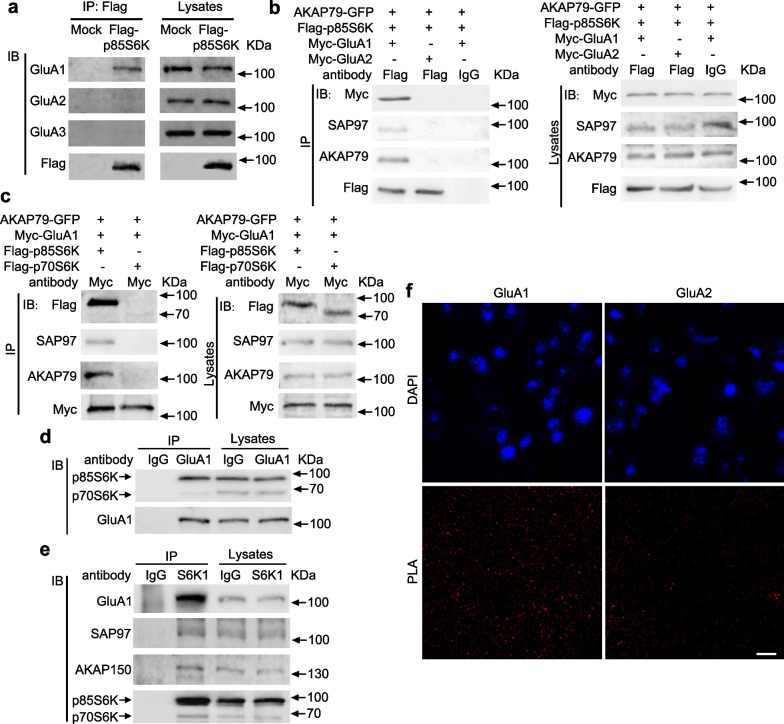
p85S6K interacts with GluA1 specifically. **a** Co-immunoprecipitation of AMPA receptors in hippocampal tissue lysates with Flag-p85S6K or empty vector (Mock) from HEK293 cell lysates. **b** Co-immunoprecipitation of myc-GluA1 or myc-GluA2 with Flag-p85S6K, with AKAP79 co-transfected in HEK293 cells. **c** Co-immunoprecipitation results for Flag-p85S6K or Flag-p70S6K with myc-GluA1, with AKAP79 co-transfected in HEK293 cells. **d**, **e** Co-immunoprecipitation of endogenous GluA1 with p85S6K in the PSD-1 fraction. **f** Confirmation of such interaction in vivo by in situ PLA. Scale bar, 10 μm. Representative blots or images of at least 3 independent experiments are shown. *IP* immunoprecipitation, *IB* immunoblot

AMPA receptors localized in PSDs play important roles in cognition by determining the efficiency of synaptic transmission and synaptic plasticity [33–35]. Thus, we tested whether p85S6K physically interacts with AMPA receptors, focusing on GluA1-GluA3 subunits [35, 36]. HEK293 cells were transfected with plasmids encoding Flag-p85S6K and the cell extracts were mixed with protein lysates from hippocampus for immunoprecipitation [37]. p85S6K was co-immunoprecipitated with the GluA1 subunit of AMPA receptor but not with GluA2 or GluA3 subunit (Fig. 2a). Then we tried to validate such interaction in HEK293 cells by exogenously overexpressing Flag-p85S6K and Myc-GluA1. However, we could not immunoprecipitate them together (Additional file 1: Fig. S4a). As p85S6K is a serine–threonine kinase, we speculated that it may serve to phosphorylate GluA1 like PKA. GluA1 binds to SAP97 to link with AKAP79/150, which anchors PKA to phosphorylate GluA1 [38–40]. As AKAP79 is expressed at a very low level whereas SAP97 is highly expressed in HEK293 cells (Additional file 1: Fig. S4b, c), we overexpressed AKAP79 with Flag-p85S6K and Myc-GluA1, and found that p85S6K was immunoprecipitated with GluA1; meanwhile, AKAP79 and SAP97 were detected in the complex (Fig. 2b, c). These results indicate that p85S6K interacts with GluA1 through AKAP79 and SAP97. Next, we investigated whether p85S6K affects the interaction between PKA and GluA1. Co-transfection of GluA1 and AKAP79 allowed the immunoprecipitation of PKA with GluA1 (Additional file 1: Fig. S5a). Overexpression of p85S6K slightly increased the immunoprecipitated PKA in GluA1 complex, but the difference was not significant (Additional file 1: Fig. S5a, b), demonstrating that p85S6K generally does not affect the interaction between PKA and GluA1. Moreover, we further confirmed that myc-GluA2 was not co-immunoprecipitated with Flag-p85S6K (Fig. 2b), and that myc-GluA1 and Flag-p70S6K could not be co-immunoprecipitated (Fig. 2c). The endogenous binding of p85S6K and GluA1 was also confirmed by co-immunoprecipitation in PSD preparations from hippocampus (Fig. 2d, e). In addition, such interaction was confirmed in vivo by in situ PLA (Fig. 2f), although the involvement of p70S6K could not be completely excluded because the antibody recognizes both p85S6K and p70S6K.

### p85S6K phosphorylates GluA1 at Ser845 and modulates synaptic GluA1

**Fig. 3:**
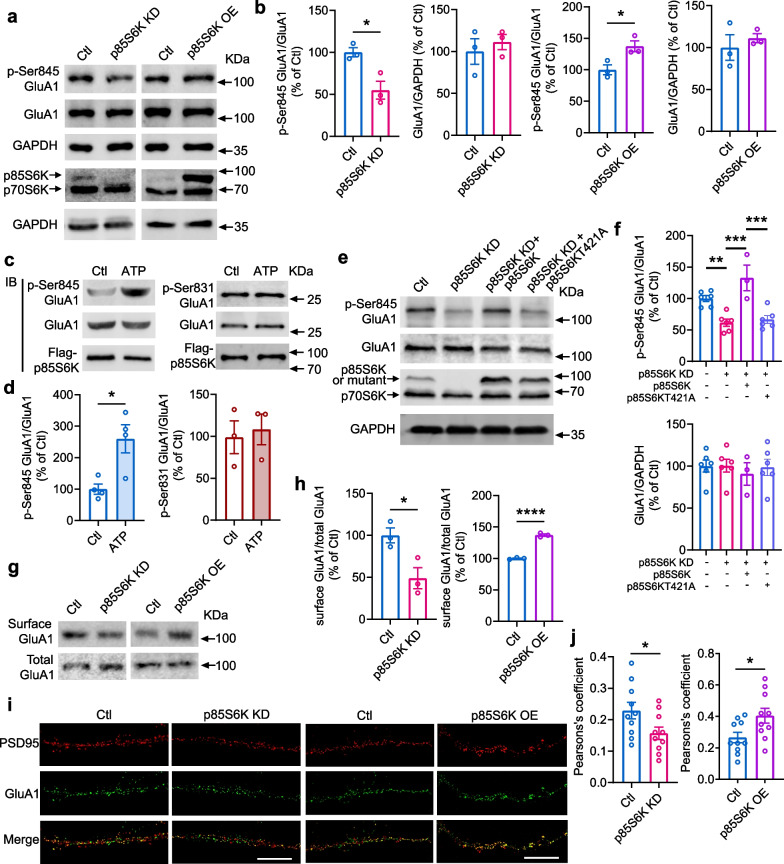
p85S6K phosphorylates GluA1 at Ser845 and enhances synaptic GluA1. **a**, **b** Knockdown of p85S6K decreased while overexpression of p85S6K increased phosphorylation of GluA1 at Ser845 (p-Ser845 GluA1) in primary hippocampal neurons. Control neurons were infected with lentiviruses expressing control siRNA or empty vectors. *n* = 3 independent experiments. *t* = 3.755, df = 4, *P* = 0.0199;* t* = 0.6291, df = 4, *P* = 0.5634; *t* = 3.234, df = 4, *P* = 0.0318; *t* = 0.6827, df = 4, *P* = 0.5323 (**b**, from left to right panels). **c**, **d** In vitro phosphorylation of GluA1 at Ser845 and Ser831 by p85S6K. *n* = 4 independent experiments for Ser845 and 3 for Ser831. *t* = 3.364, df = 6, *P* = 0.0151 (**d**, left panel) and Mann–Whitney *U* = 4, *P* > 0.9999 (**d**, right panel). **e**, **f** Overexpression of catalytically inactive p85S6KT421A did not rescue the decreased phosphorylation of GluA1 at Ser845 induced by knockdown of p85S6K. *n* = 3–6 independent experiments. *F* = 14.52, *P* < 0.0001. **g**, **h** Knockdown of p85S6K decreased while overexpression of p85S6K increased surface GluA1 in primary hippocampal neurons. *n* = 3 independent experiments. *t* = 3.312, df = 4, *P* = 0.0296 (**h**, left panel) and *t* = 18.96, df = 4, *P* < 0.0001 (**h**, right panel). **i**, **j** Colocalization of GluA1 and PSD95 in primary hippocampal neurons overexpressing p85S6K. Scale bar: 10 μm. *n* = 10 neurons per group. *t* = 2.168, df = 18, *P* = 0.0438 (**j**, left panel) and *t* = 2.412, df = 18, *P* = 0.0268 (**j**, right panel). Data are presented as mean ± SEM. Unpaired *t* test, two-tailed (**b;** left panel in **d**; **h**; and **j**), Mann–Whitney test, two-tailed (**d**, right panel) and ordinary one-way ANOVA followed by Tukey's test (**f**). *P < 0.05, **P < 0.01, ***P < 0.001, ****P < 0.0001

The above results demonstrate that like PKA, p85S6K forms a complex with GluA1. PKA phosphorylates GluA1 at Ser845 to potentiate its response to glutamate [41]. To further examine the function of p85S6K, the expression level of p85S6K was manipulated in primary neurons (Fig. 3a). As expected, the phosphorylation of GluA1 at Ser845 was reduced by knockdown of p85S6K and enhanced by overexpression of p85S6K (Fig. 3a, b). Furthermore, in vitro phosphorylation assay demonstrated that p85S6K directly phosphorylated GluA1 at Ser845 (Fig. 3c, d), and that phosphorylation of GluA1 at Ser831 was not affected by p85S6K (Fig. 3c, d), indicating that Ser831 was not involved in the effect of p85S6K. To further demonstrate the role of kinase activity of p85S6K in GluA1 phosphorylation, the most important residue Thr421 of p85S6K (the same to Thr389 in p70S6K) for its catalytic activity was mutated to Ala (p85S6KT421A). The mutant did not reverse the decrease of Ser845 phosphorylation of GluA1 induced by p85S6K knockdown; however, re-introduction of the wild-type p85S6K rescued the Ser845 phosphorylation (Fig. 3e, f). These data demonstrated that the catalytic activity of p85S6K plays a critical role in GluA1 phosphorylation.

Phosphorylation of GluA1 at Ser845 potentiates membrane insertion and synaptic delivery of GluA1 [42]. A recent study showed that phosphorylation of GluA1 at Ser845 also plays a role in blocking GluA1 internalization [43]. This trafficking of GluA1 has been associated with synaptic plasticity, which underlies the cognitive function [44]. Thus, we investigated whether surface and synaptic GluA1 are affected by p85S6K. In concordance with what we observed for GluA1 phosphorylation, knockdown of p85S6K reduced, while overexpression enhanced surface GluA1 in primary cultured neurons (Fig. 3g, h). Furthermore, knockdown of p85S6K decreased, while overexpression of p85S6K increased the colocalization of GluA1 and PSD95 in neurons (Fig. 3i, j), indicating increase of synaptic GluA1-containing AMPA receptors.

**Fig. 4:**
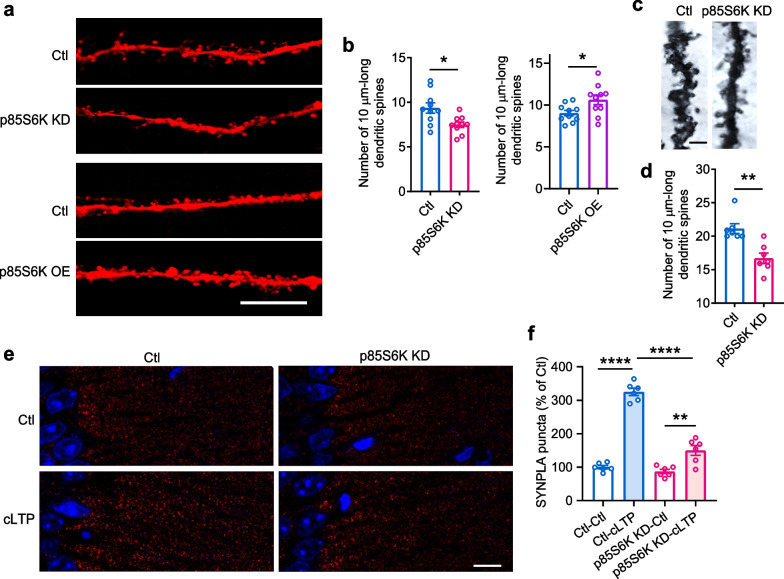
p85S6K promotes spine density and synaptic strength. **a**, **b** Spine density in primary cultured hippocampal neurons after knockdown or overexpression of p85S6K. Scale bar, 10 μm. *n* = 10 neurons per group. *t* = 2.873, df = 18, *P* = 0.0101 (**b**, left panel); *t* = 2.406, df = 18, *P* = 0.0271(**b**, right panel). **c**, **d** Spine density in the hippocampus of WT mice after knockdown of p85S6K. Scale bar, 1 μm. *n* = 7 slices from 3 mice per group. Mann–Whitney *U* = 1.500, *P* = 0.0023. **e**, **f** SYNPLA signals after cLTP in hippocampus with p85S6K knockdown. Scale bar, 10 μm. *n* = 6 slices from 3 mice per group. *F*_(1, 20)_ = 203.8, *P* < 0.0001 for cLTP induction effect; *F*_(1, 20)_ = 87.13, *P* < 0.0001 for p85S6K knockdown effect. Data are presented as mean ± SEM. Unpaired *t* test, two-tailed (**b**), Mann–Whitney test, two-tailed (**d**) and two-way ANOVA followed by Tukey's test (**f**). *P < 0.05, **P < 0.01, ****P < 0.0001. *Ctl* control, *KD* knockdown

Dendritic spines, which hold the synapses, are closely correlated with surface GluA1 [45, 46]. Thus, the spine density may be influenced by p85S6K. Knockdown of p85S6K decreased the spines in primary neurons, whereas overexpression of p85S6K increased the spine density (Fig. 4a, b). Further, Golgi staining showed that the number of spines was decreased in the hippocampus with p85S6K knockdown (Fig. 4c, d). Then SYNPLA, which detects synaptic insertion of GluA1-containing AMPA receptors [26], was recorded in the hippocampal neurons from acute brain slices of mice. Chemical treatment for LTP induction increased SYNPLA signal significantly (Fig. 4e, f). When p85S6K was knocked down, the SYNPLA signal was reduced (Fig. 4e, f). Taken together, these results indicate a critical role for p85S6K in the establishment of functional synapses.

### p85S6K expression is decreased in brains from AD patients and AD model mice

**Fig. 5:**
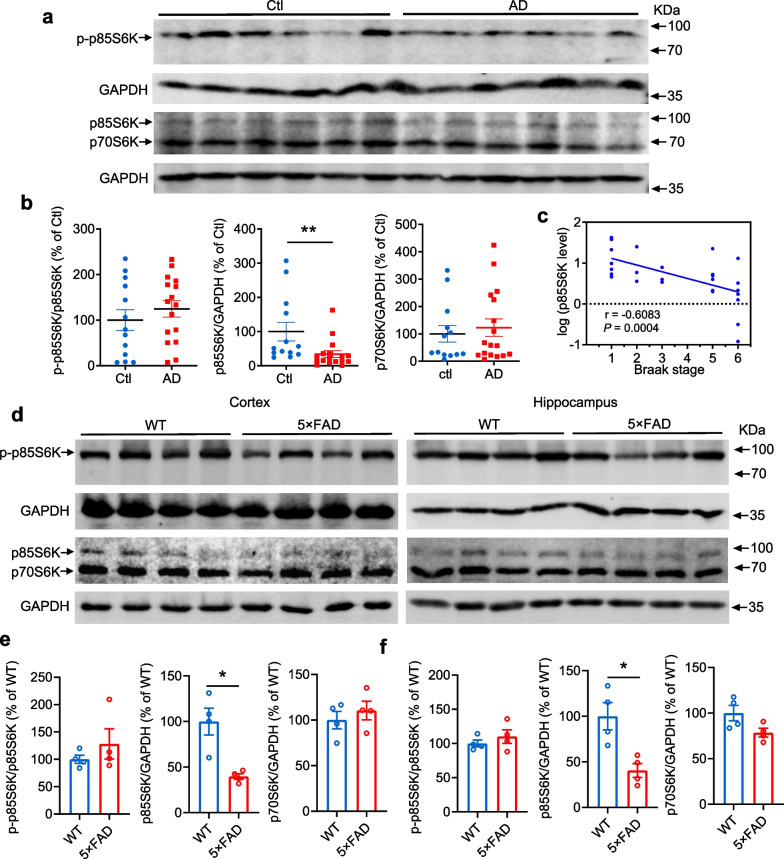
p85S6K expression is specifically decreased in human AD brains and  5×FAD mouse brains. **a**, **b** Expression of p85S6K/p70S6K and phosphorylated p85S6K in P2 pellets from fractionation of postmortem temporal cortex from human AD brains and non-demented control (Ctl). *n* = 13 for Ctl and *n* = 17 for AD. *t* = 0.8582, df = 27, *P* = 0.3984 for p-p85S6K, Mann–Whitney *U* = 39, *P* = 0.0021 for p85S6K, Mann–Whitney *U* = 107, *P* = 0.9016 for p70S6K. **c** Pearson correlation of Braak stage and the expression level of p85S6K in human brains. **d–f** Expression of p85S6K/p70S6K and phosphorylated p85S6K in P2 pellets from fractionation of cortex (**d**, **e**) and hippocampus (**d**, **f**) from 7-month-old  5×FAD mice. *n* = 4 mice per group. *t* = 0.9933, df = 6, *P* = 0.3589; *t* = 4.005, df = 6, *P* = 0.0238; *t* = 0.7484, df = 6, *P* = 0.4825 (**e**, from left to right panels). *t* = 0.9035, df = 6, *P* = 0.4011;* t* = 3.548, df = 6, *P* = 0.0121; *t* = 2.221, df = 6, *P* = 0.0681 (**f**, from left to right panels). Data are presented as mean ± SEM. Mann–Whitney test, two-tailed (**b,** middle and right panels) and unpaired *t* test, two-tailed (**b**, left panel; and **e**, **f**). *P < 0.05, **P < 0.01

As p85S6K was shown to be important for functional synapses and contribute to cognitive function, we measured its expression in the P2 fraction (crude synaptosomes) of human AD brains to further explore whether it was affected in AD. p85S6K was significantly decreased in the P2 fraction of the postmortem AD brains compared with non-demented control brains (Fig. 5a, b; see Additional file 1: Fig. S6 for additional blots). Furthermore, the p85S6K protein level negatively correlated with Braak stage (Fig. 5c). Moreover, the amount of p85S6K in P2 fraction was decreased in hippocampus and cortex of  5×FAD mice (Fig. 5d–f), which exhibit patterns most similar to AD [47]. But p85S6K was not reduced in the P2 fraction of cortex of another AD model—APP/PS1 mice (Additional file 1: Fig. S7). The phosphorylated p85S6K level was not significantly altered in P2 fraction in both AD patients and  5×FAD mice (Fig. 5a, b, d, e, f), indicating that the phosphorylation level of p85S6K was not changed in AD. The reduced expression of p85S6K was also observed in the PSD-1 fraction of cortex from  5×FAD mice and APP/PS1 mice (Additional file 1: Fig. S8). To better reflect the role of p85S6K in synapses, the expression of PSD95 was measured. The expression level of PSD95 in P2 pellets from postmortem AD brains was comparable to that of non-demented controls (Additional file 1: Fig. S9a, b). Similar expression patterns of PSD95 were observed in the hippocampus and cortex of  5×FAD mice (Additional file 1: Fig. S9c, d). Correspondingly, the expression of PSD95 was not altered significantly in the PSD-1 fraction of cortex of  5×FAD or APP/PS1 mice (Additional file 1: Fig. S8). These results indicate that PSD95 expression is not significantly altered in the synaptosomal compartment. The reduction of p85S6K in the pathological process of AD did not parallel PSD95.

### Overexpression of p85S6K ameliorates cognitive deficits in AD mice

**Fig. 6:**
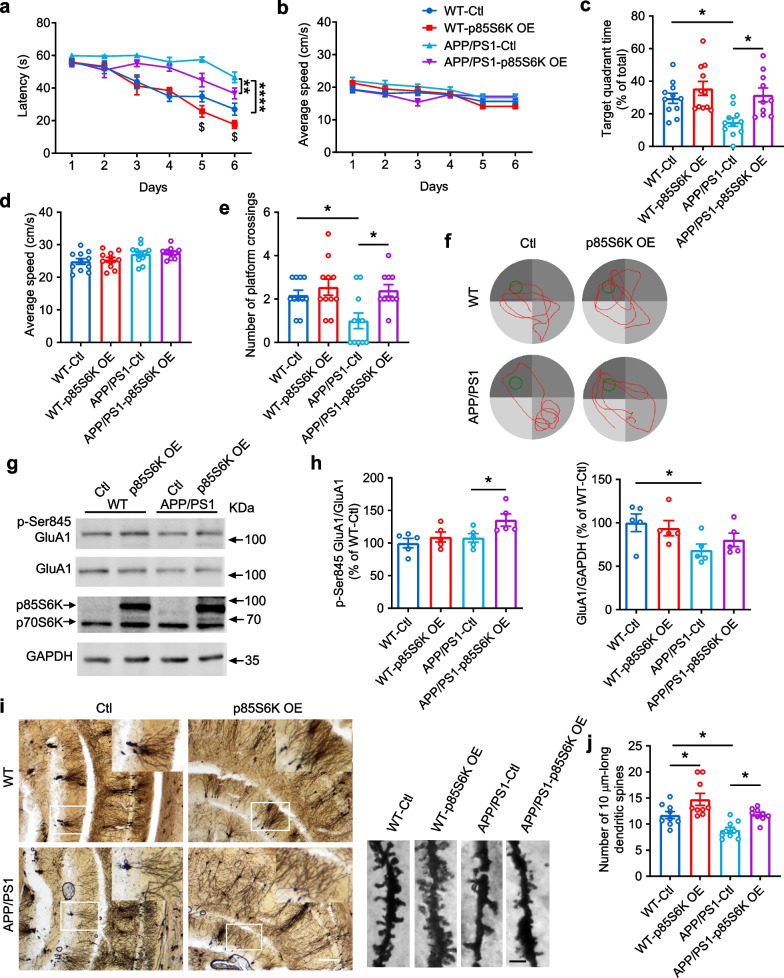
Overexpression of p85S6K ameliorates cognitive decline and spine deficits in AD mice. **a–f** Morris water maze was carried out to assess spatial learning and memory. **a** Latency of WT and APP/PS1 mice to locate the hidden platform in the training period. *F*_(1, 39)_ = 97.50, *P* < 0.0001 for genotype effect, *F*_(1, 39)_ = 14.48, *P* = 0.0005 for p85S6K overexpression effect. **b** The average speed of WT and APP/PS1 mice in the training period. **c** The time spent in the target quadrant in the probe test. *F*_(1, 39)_ = 6.731, *P* = 0.0133 for genotype effect, *F*_(1, 39)_ = 9.915, *P* = 0.0013 for p85S6K overexpression effect. **d** The average speed of WT and APP/PS1 mice in the probe test. **e** The number of platform crossings of WT and APP/PS1 mice in the probe test. *F*_(1, 39)_ = 4.522, *P* = 0.0398 for genotype effect, *F*_(1, 39)_ = 7.984, *P* = 0.0074 for p85S6K overexpression effect. **f** Representative swimming trajectories in the probe test. The green circle represented the location of the hidden platform. *n* = 11 mice for WT-Ctl, WT-p85S6K OE, and APP/PS1-Ctl groups; *n* = 10 for the APP/PS1-p85S6K OE group. **g**, **h** The expression levels of p-Ser845 GluA1 and total GluA1 in the hippocampus after injection of AAVs expressing p85S6K or control AAVs. *n* = 5 mice per group. Left panel in **h**: *F*_(1, 16)_ = 4.731, *P* = 0.0450 for genotype effect, *F*_(1, 16)_ = 5.540, *P* = 0.0317 for p85S6K overexpression effect; right panel in **h**: *F*_(1, 16)_ = 6.906, *P* = 0.0183 for genotype effect, *F*_(1, 16)_ = 0.1139, *P* = 0.7401 for p85S6K overexpression effect. **i**, **j** Spine density in the hippocampus of WT and APP/PS1 mice after overexpression of p85S6K. Scale bars, 200 μm (left panel) and 1 μm (right panel). *n* = 9 slices from 4 mice per group. *F*_(1, 32)_ = 14.97, *P* = 0.0005 for genotype effect, *F*_(1, 32)_ = 16.22, *P* = 0.0003 for p85S6K overexpression effect. Data are presented as mean ± SEM. Three-way ANOVA (**a**, **b**) or two-way ANOVA (**c**, **d**, **e**, **h**, **j**) followed by Tukey's or Bonferroni's test. *P < 0.05, **P < 0.01, ****P < 0.0001.  *Ctl* control, i.e., mice injected with control AAVs. *OE* overexpression

Next, we investigated whether p85S6K could ameliorate the cognitive deficits in AD mice. As  5×FAD mice exhibit higher anxiolytic behavior [48], we used APP/PS1 mice for behavior examination. Like the knockdown of p85S6K, overexpression of p85S6K (Fig. 6g) did not affect the locomotor activity or anxiety-like behavior (Additional file 1: Fig. S10). When p85S6K was overexpressed, APP/PS1 mice were able to locate the hidden platform faster during the spatial learning in MWM (Fig. 6a). Moreover, WT mice were able to locate the hidden platform faster on day 5 and day 6 when p85S6K was upregulated (Fig. 6a). In the probe test, the time spent in the target quadrant and the number of platform crossings were significantly increased by p85S6K overexpression (Fig. 6c, e, f). The swimming speed did not differ significantly (Fig. 6b, d). Further, the expression level of GluA1 was decreased in APP/PS1 mice compared to wild-type mice (Fig. 6g, h), and was not significantly altered by overexpression of p85S6K. However, overexpression of p85S6K increased the phosphorylation of GluA1 at Ser845 in APP/PS1 mice (Fig. 6g, h). Moreover, the neuronal dendrites in the CA1 region of APP/PS1 mice showed an obvious decline in spine density compared to that of WT mice (Fig. 6i, j), and this decline was significantly prevented by overexpression of p85S6K (Fig. 6i, j). In addition, the spine density in WT mice was enhanced by overexpression of p85S6K (Fig. 6i, j). These results demonstrate that overexpression of p85S6K can ameliorate the synaptic and cognitive impairment in AD.

As overexpression of p85S6K also showed beneficial effects in WT mice, we overexpressed p85S6K in S845A mice, in which phosphorylation of GluA1 at Ser845 was deficient due to its mutation to alanine. In these mice, p85S6K overexpression had no effect on spatial learning and memory (Additional file 1: Fig. S11a–f), nor did it increase the spine density (Additional file 1: Fig. S11g, h). These data further confirm that p85S6K exerts its effect through phosphorylation of Ser845 of GluA1.

## Discussion

S6K1-knockout mice display early-onset contextual fear memory deficits and impaired MWM acquisition [49]. But the contribution of p70S6K and p85S6K to the memory is hard to separate. In the current study, we demonstrated that p70S6K and p85S6K were distributed differentially, with p85S6K more enriched in PSDs and p70S6K mainly present in the compartments other than PSDs. Furthermore, we illustrated that p85S6K, but not p70S6K, formed a complex with GluA1 via AKAP79/150 and SAP97, which allowed it to phosphorylate GluA1 at Ser845 and promote GluA1 synaptic incorporation. Moreover, we uncovered the role of p85S6K in synaptic function and cognition, as demonstrated by spine deficits and impaired memory caused by p85S6K knockdown and improved cognitive deficits in AD model mice by p85S6K overexpression. Notably, p85S6K was decreased in crude synaptosomal preparations from brains of AD patients and  5×FAD mice. These results suggest a working model where p85S6K present in PSDs may have a significant impact on functional synapses and consequent cognition, while downregulation of p85S6K in AD can diminish the functional synapses and thus aggravate cognitive impairment.

Activation of p70S6K mainly occurs along dendritic shafts responsible for fast dendritic translation, with much less localization in dendritic spines [20]. Our PSD fractionation and synaptosome trypsin cleavage assay also indicated that p70S6K was less distributed in PSDs, and even less with its more abundant expression than p85S6K. Besides the 23 amino acids in the N-terminus of p85S6K which are predicted to be a NLS sequence, an additional NLS signal of 9 amino acids has also been found near the C-tail of p85S6K (www.csbio.sjtu.edu.cn/bioinf/INSP/, Additional file 1: Fig. S12) [50]. This may explain why p70S6K can also be found to locate in nucleus. A six-tandem arginine repeat (6R motif) is an important component for NLS as shown in Additional file 1: Fig. S12. However, proteins or peptides harboring an arginine-rich region are also able to penetrate the mammalian cell membrane [51, 52]. As such, p85S6K has recently been recognized as a secreted protein in cancer cells [3]. Furthermore, arginine-rich helix is found to be present in transmembrane proteins such as potassium channel KvAP and assist in membrane anchoring [53, 54]. Thus, 6R motif may play a role in p85S6K anchoring in PSDs.

Both PKA and p85S6K belong to the AGC kinase family and are serine–threonine kinases. Our finding that both SAP97 and AKAP79 were present in the p85S6K-GluA1 complex indicated that p85S6K forms a complex with GluA1 similar as PKA does, which binds with GluA1 through AKAP79 and SAP97 and thereby phosphorylates GluA1 at Ser845 [39]. As AKAP79 is a signaling scaffold protein binding to kinases and phosphatases [55] and determines the localization of PKA [56], AKAP79 may be also involved in translocation of p85S6K to the PSDs and allow p85S6K to phosphorylate GluA1. Our data further indicated that PKA and p85S6K do not compete for interacting with GluA1. Thus, PKA and p85S6K may bind AKAP79 at different domains. Considering that p70S6K does not interact with GluA1, the N-terminus of p85S6K probably plays a role in its AKAP79 binding.

AMPA receptors mediate a majority of fast excitatory synaptic transmission and are important for synaptic plasticity and memory [57, 58]. The functions of AMPA receptors are prominently controlled by phosphorylation of the subunit [57]. For GluA1, phosphorylation at Ser845 promotes GluA1 targeting to the cell surface and synapses or reduces the internalization of GluA1, which usually occurs in synaptic plasticity and learning and memory processes. The present study demonstrated that knockdown of p85S6K reduced GluA1 phosphorylation while overexpression of p85S6K enhanced phosphorylation. Meanwhile, the cell-surface and synaptic GluA1 are regulated correspondingly, indicating that p85S6K contributes to sustaining synaptic incorporation of GluA1 by maintaining its phosphorylation. Further, our data indicated a possible increase of Ca^2+^-permeable GluA1 homomers (CP-AMPARs) by p85S6K. Elevated surface CP-AMPARs have been shown to facilitate LTP and improve hippocampus-dependent spatial learning [24]. The expression of GluA1 is down-regulated in the brains of AD patients and transgenic AD mice [59, 60]. Examination of GluA1 in P2 pellets also showed that GluA1 expression was reduced in  5×FAD and APP/PS1 mice (Additional file 1: Fig. S13). Risk factors such as β-amyloid can affect the phosphorylation of GluA1 at Ser845 and surface level of GluA1, causing reduction of surface CP-AMPARs or abnormal synaptic CP-AMPAR incorporation [24, 61, 62]. Our study demonstrated that overexpression of p85S6K increased surface GluA1 via phosphorylation of GluA1 at Ser845 and improved learning and memory in AD mice, implicating that p85S6K may restore the CP-AMPARs in AD. Correspondingly, the density of dendritic spines was also modified by p85S6K without alterations of the level of GluA1 (Figs. 3a, b, 6g, h). These data suggest that the modulation of GluA1 phosphorylation by p85S6K leads to quantitative changes of spines. Therefore, p85S6K plays an important role in GluA1 phosphorylation and trafficking, thus contributing to synaptic function and cognition.

The expression of p70S6K is reported to be elevated in the whole homogenates of AD brains [19]. Most studies only measured the expression level of p70S6K with only one study showing increased p85S6K at the same time [19]. Here we fractionated the crude synaptosomal compartment of the cortex and demonstrated decreased distribution of p85S6K in the synaptosomal compartments in AD brains, whereas the alteration of p70S6K is not obvious. These data suggest that p85S6K and p70S6K may have different alterations in different cellular compartments in AD. It has been shown that p70S6K plays a critical role in protein translation during long-term synaptic potentiation and memory [16, 17, 20]. However, the present study showed that the overexpression or knockdown of p85S6K had little effect on the expression level of GluA1 (Figs. 3a, b, 6g, h). Taken together, p85S6K may be preferentially involved in synaptic regulation, while p70S6K is mainly involved in other effects including protein synthesis.

Similar to p70S6K, mTOR is the defined upstream regulator of p85S6K. mTOR signaling has been shown to be implicated in various brain functions [9, 10]. The mTOR/p70S6K axis has been shown to mainly modulate synaptic expression of GluA1 via protein translation [63–65]. The present study suggests that mTOR may modulate synaptic targeting of GluA1 through p85S6K in addition to p70S6K. Lines of evidence indicate that mTOR/p70S6K contributes to the AD pathogenesis by influencing β-amyloid peptide production and degradation as well as tau hyperphosphorylation [11, 66, 67]. Our study demonstrated that p85S6K is distributed and functions differentially. In addition, p70S6K and p85S6K show different alterations in the synaptosomal compartments of AD brains, suggesting that they are differentially affected by AD pathology. As the cognitive performance of AD mouse models has a strong inverse correlation with mTOR/p70S6K signaling [12] and although inhibition of mTOR/p70S6K improves memory deficits and delays pathological progression in AD mice [67–69], mTOR/p70S6K is also required for synaptic function and memory [13, 70], moderate inhibition of mTOR/p70S6K signaling for AD treatment or prevention may be appropriate [71]. Therefore, steering the translation of S6K1 to p85S6K may be particularly relevant.

The present study further showed that the reduction of p85S6K did not parallel PSD95 in AD brains. Measurements of PSD95 in AD have produced contradictory results in both animal and human brains [72]. Some studies showed decreased whereas others showed increased or unchanged expression level of PSD95 in AD brains compared to controls [72, 73]. Multiple mechanisms have been proposed to explain this discrepancy, including reactive or compensatory expression [72]. Therefore, the downregulation of p85S6K but not PSD95 may indicate that p85S6K is more important in the maintenance of functional synapses in AD, making it an appealing therapeutic target.

Besides AMPA receptors, NMDA receptors are also located in PSDs and play important roles in cognition [74]. The function of NMDA receptors can also be regulated by phosphorylation [75]. In the present study, p85S6K was found to be enriched in PSDs, thus may also phosphorylate and influence the function of NMDA receptors. Whether p85S6K modulates the function of NMDA receptors warrants further investigations.

## Conclusions

In summary, the present study illustrates the interaction between p85S6K and GluA1 and the regulatory role of p85S6K in synaptic function and cognition, which can advance our understanding of modulation of GluA1-containing AMPA receptors. Furthermore, we found pathological alterations of p85S6K in AD. The present study provides a new mechanism of the role for S6K1 in cognition and uncovers p85S6K-GluA1 signaling in memory modulation.

## Supplementary Information

**Additional file 1. Table S1**: Information of brain donors in this study. **Fig. S1**: The expression pattern of S6K1 in HEK293 cells and neurons. **Fig. S2**: The quantification of PSD95 and synaptophysin in fractions and the validation of p70S6K antibodies. **Fig. S3**: Knockdown of p85S6K efficiently reduces p85S6K in PSD-1 and does not affect the locomotor activity and anxiety-like behavior. **Fig. S4**: p85S6K cannot be immunoprecipitated with GluA1 with little expression of AKAP79 in HEK293 cells. **Fig. S5**: p85S6K does not interfere with the interaction between GluA1 and PKA. **Fig. S6**: The immunoblots of p85S6K/p70S6K and phosphorylated form in remaining human temporal cortex samples related to Fig. 5a. **Fig. S7**: p85S6K expression in P2 pellets of cortex of APP/PS1 mice. **Fig. S8**: p85S6K expression is decreased in PSD-1 of cortex of  5×FAD mice. **Fig. S9**: PSD95 expression is not altered in AD brains. **Fig. S10**: Overexpression of p85S6K does not affect the locomotor activity and anxiety-like behavior. **Fig. S11**: Upregulation of p85S6K does not enhance the spatial learning and spine density in S845A mice. **Fig. S12**: The NLS predicted in p85S6K by www.csbio.sjtu.edu.cn/bioinf/INSP/. **Fig. S13**: The expression of GluA1 and its phosphorylation at Ser845 are reduced in P2 pellets from fractionation of cortex of 7-month old  5×FAD and 9-month old APP/PS1 mice.

## Data Availability

The datasets used and/or analyzed are available from the corresponding author on reasonable request.

## Abbreviations

S6K1: Ribosomal protein S6 kinase 1
PKA: Protein kinase A
mTOR: Mammalian target of rapamycin
NLS: Nuclear localization signal
AD: Alzheimer’s disease
PSD: Postsynaptic density
SAP97: Synapse-associated protein 97
AKAP79/150: A-kinase anchoring protein 79/150
PLA: Proximity ligation assay
SYNPLA: Synaptic PLA
LTP: Long-term potentiation
MWM: Morris water maze
NOR: Novel object recognition
CP-AMPAR: Ca^2+^-permeable GluA1 homomer
