# Supplementary material for: p85S6K sustains synaptic GluA1 to ameliorate cognitive deficits in Alzheimer’s disease
Source: Transl Neurodegener. 2023 Jan 9;12:1. doi: 10.1186/s40035-022-00334-w (PMC9827685; doi:10.1186/s40035-022-00334-w)
Supplement: Supplementary file 1 — Additional file 1. Table S1: Information of brain donors in this study. Fig. S1: The expression pattern of S6K1 in HEK293 cells and neurons. Fig. S2: The quantification of PSD95 and synaptophysin in fractions and the validation of p70S6K antibodies. Fig. S3: Knockdown of p85S6K efficiently reduces p85S6K in PSD-1 and does not affect the locomotor activity and anxiety-like behavior. Fig. S4: p85S6K cannot be immunoprecipitated with GluA1 with little expression of AKAP79 in HEK293 cells. Fig. S5: p85S6K does not interfere with the interaction between GluA1 and PKA. Fig. S6: The immunoblots of p85S6K/p70S6K and phosphorylated form in remaining human temporal cortex samples related to Fig. 5a. Fig. S7: p85S6K expression in P2 pellets of cortex of APP/PS1 mice. Fig. S8: p85S6K expression is decreased in PSD-1 of cortex of 5×FAD mice. Fig. S9: PSD95 expression is not altered in AD brains. Fig. S10: Overexpression of p85S6K does not affect the locomotor activity and anxiety-like behavior. Fig. S11: Upregulation of p85S6K does not enhance the spatial learning and spine density in S845A mice. Fig. S12: The NLS predicted in p85S6K by www.csbio.sjtu.edu.cn/bioinf/INSP/. Fig. S13: The expression of GluA1 and its phosphorylation at Ser845 are reduced in P2 pellets from fractionation of cortex of 7-month old 5×FAD and 9-month old APP/PS1 mice. [file 40035_2022_334_MOESM1_ESM.pdf]

Supplementary Information

p85S6K sustains synaptic GluA1 to ameliorate cognitive deficits in Alzheimer's disease

Jia-Bing Li, Xiao-Yu Hu, Mu-Wen Chen, Cai-Hong Xiong, Na Zhao, Yan-hui Ge, Hao Wang, Xiao-Ling Gao, Nan-Jie Xu, Lan-Xue Zhao, Zhi-hua Yu, Hong-Zhuan Chen*, Yu Qiu*

Supplementary Table

Table S1 Information of brain donors in this study

Diagnosis	Non-demented control(n=13)	Alzheimer's disease(n=17)
Age (Year)		
Mean ± SD	76.36±10.94	76.76±8.33
Range	60-96	61-88
Gender		
Male	3	6
Female	10	11
APOE		
APOE3	5	9
APOE4	8	8
Braak		
Mean ± SD	1.64±1.21	5.53±0.51
Range	1-5	5-6
PMD(h)		
Mean ± SD	6.08±1.25	5.07±0.85
Range	4.17-8.42	3.83-6.42
Brain weight(kg)		
Mean ± SD	1.18±0.122	1.04±0.146
Range	0.978-1.429	0.835-1.355

Supplementary Figures and Figure legends

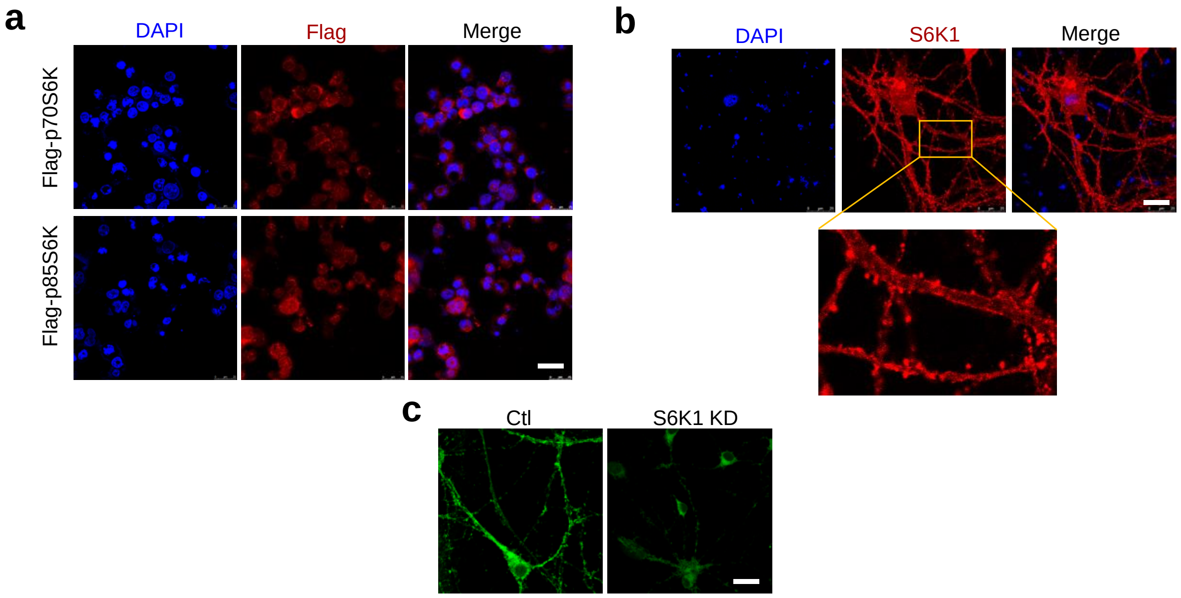

Fig. S1 The expression pattern of S6K1 in HEK293 cells and neurons. **a** The expression pattern of exogenously expressed p85S6K and p70S6K in HEK293 cells. **b** The expression pattern of endogenous S6K1 using the anti-p70S6K antibody which recognized both p85S6K and p70S6K in cultured neurons. **c** S6K1 was immunostained after S6K1 was knocked down by infecting cultured neurons with lentiviruses expressing S6K1 shRNA (S6K1 KD). Scale bar: 25 μm. Representative images of 3 independent experiments.

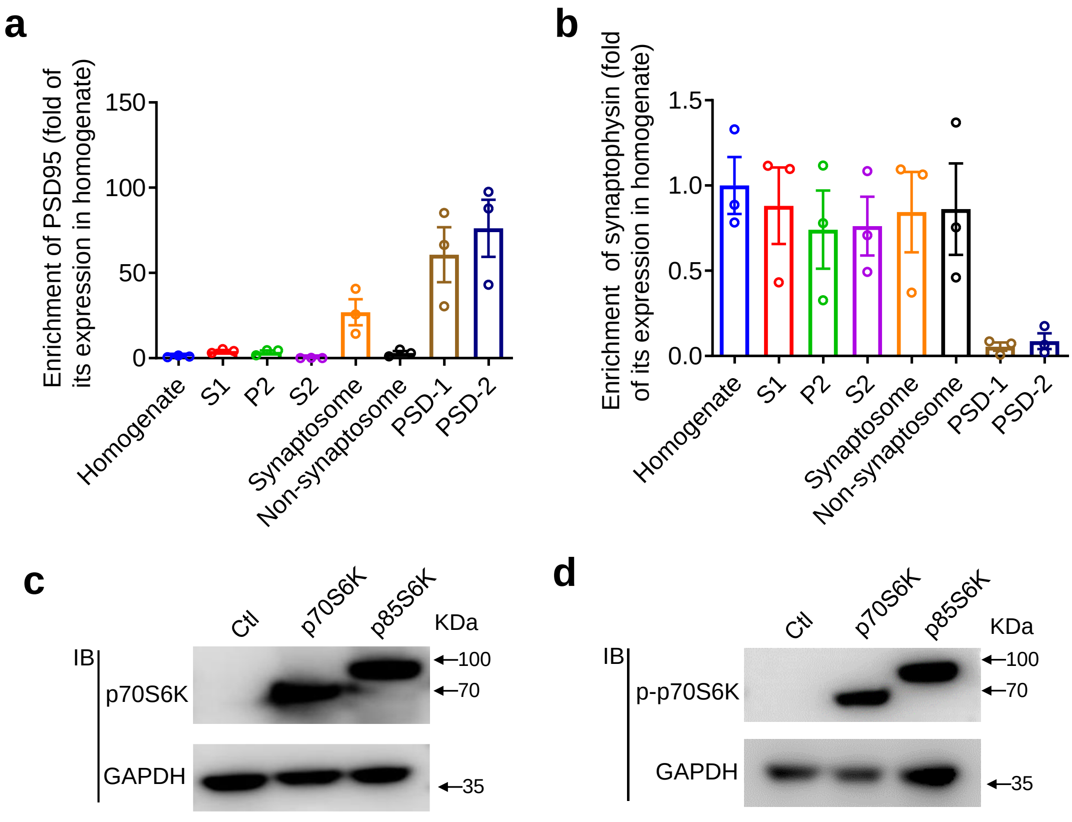

Fig. S2 The quantification of PSD95 and synaptophysin in fractions and the validation of p70S6K antibodies. **a, b** The quantification of PSD95 (a) and synaptophysin (b) in fractions shown in Fig. **1a**. **c, d** Anti-p70S6K and anti-phosphorylated p70S6K (p-p70S6K) antibodies detected p70S6K and p85S6K (c) and their phosphorylated form (d) similarly. HEK293 cells were transfected with same amounts of Flag-p70S6K and Flag-p85S6K plasmids. Data are presented as mean ± SEM. n = 3 independent experiments.

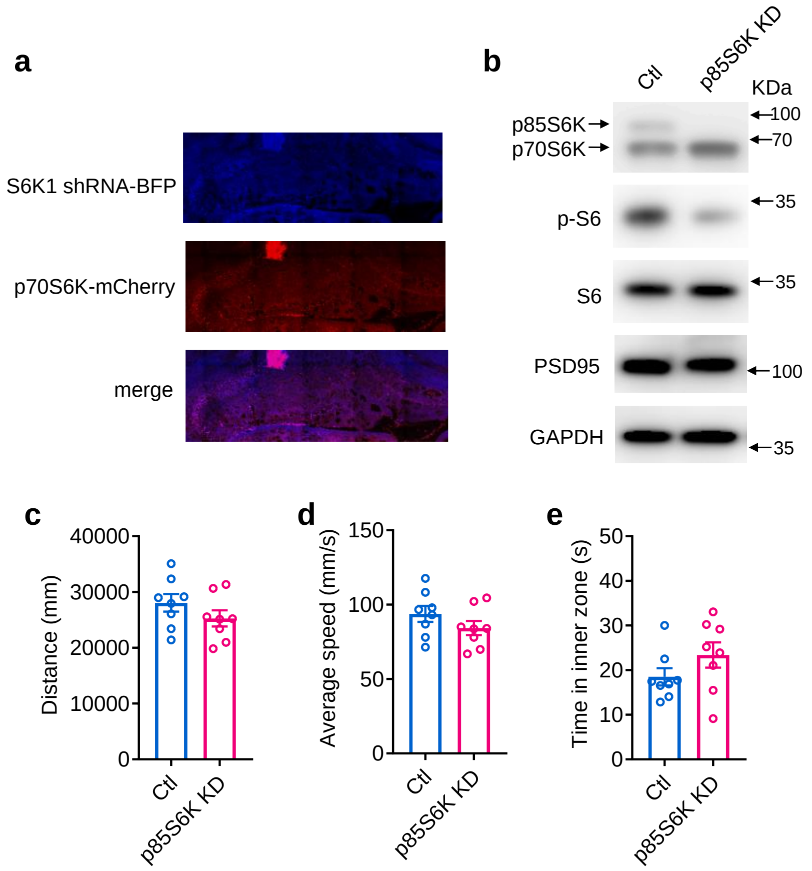

Fig. S3 Knockdown of p85S6K efficiently reduces p85S6K in PSD-1 and does not affect the locomotor activity and anxiety-like behavior. **a** Colocalization of S6K1 shRNA and p70S6K overexpression showed by their corresponding linked fluorescent proteins. **b** Knockdown of p85S6K was confirmed by its reduction of p85S6K expression and the phosphorylation of its downstream effector S6 in PSD-1. n = 3 independent experiments. p-S6: phosphorylated S6. **c** Distance moved in open field test. $t = 1.305$, df = 14, $P = 0.2129$. **d** Average speed of mice in open field test. **e** Time spent in the inner zone in open field test. $t = 1.433$, df = 14, $P = 0.1738$. n = 8 mice per group. Data are presented as mean ± SEM. Unpaired t test, two-tailed.

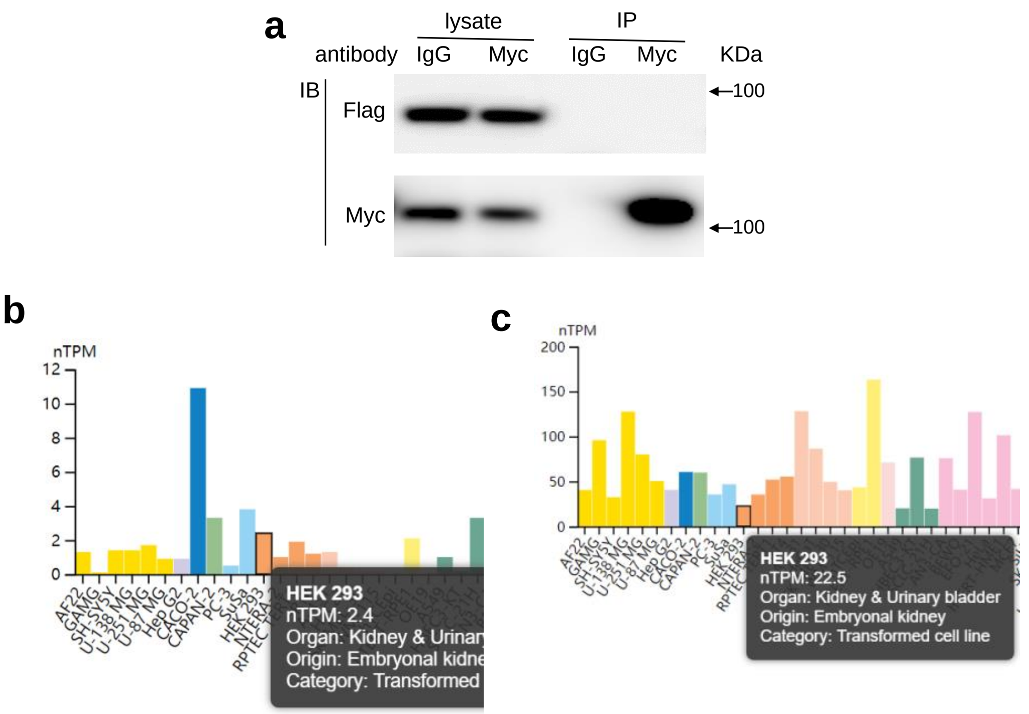

Fig. S4 p85S6K cannot be immunoprecipitated with GluA1 with little expression of AKAP79 in HEK293 cells. **a** Co-immunoprecipitation of myc-GluA1 and Flag-p85S6K failed in HEK293 cell lysates. Representative blot of at least 3 independent experiments. **b, c** The RNA expression level of AKAP79 (**b**) and SAP97 (**c**) in HEK293 cells from the Human Protein Atlas (https://www.proteinatlas.org/).

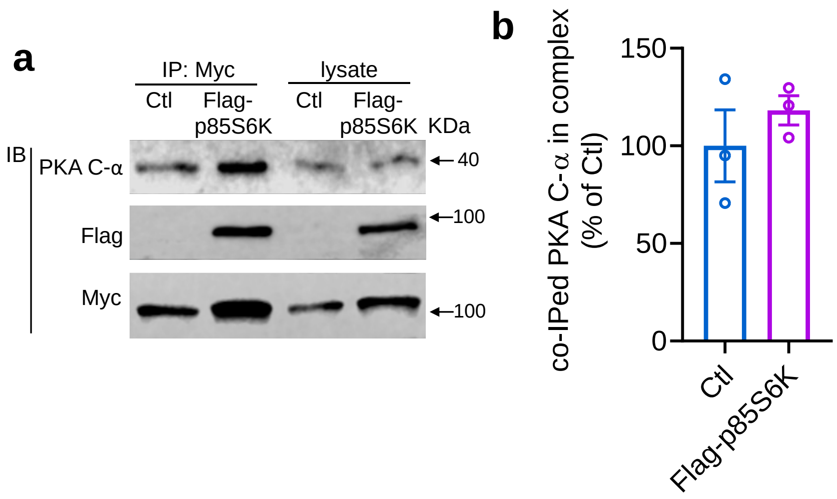

Fig. S5 p85S6K does not interfere with the interaction between GluA1 and PKA. **a, b** Immunoprecipitation of myc-GluA1 in HEK293 cells transfected myc-GluA1, AKAP79-GFP with or without Flag-p85S6K overexpression. n = 3 independent experiments. $t = 0.9139$, df = 4, $P = 0.4125$. Data are presented as mean ± SEM. Unpaired t test, two-tailed.

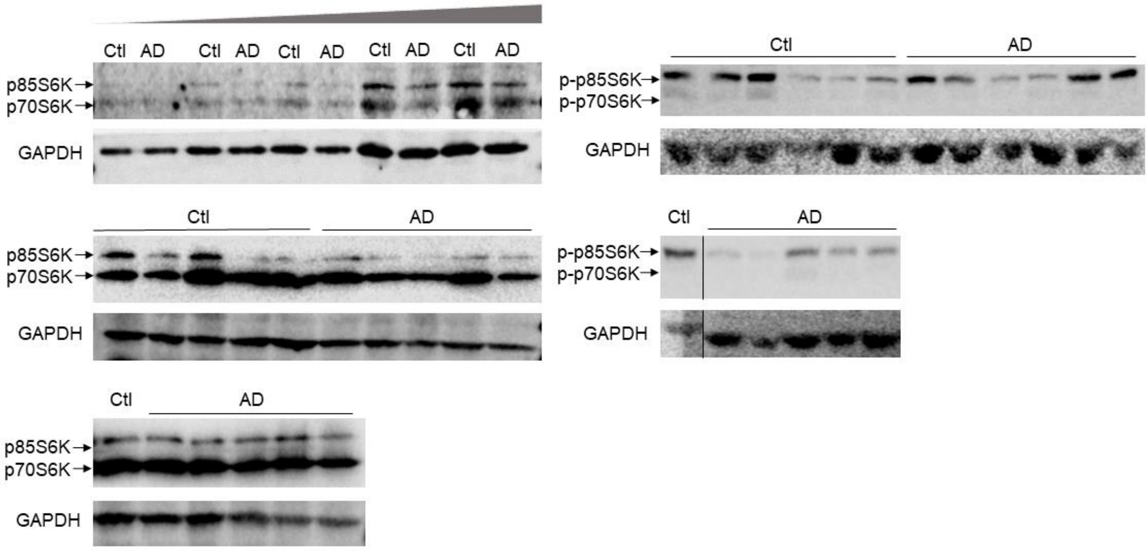

Fig. S6 The immunoblots of p85S6K/p70S6K and phosphorylated form in remaining human temporal cortex samples related to Fig. **5a**.

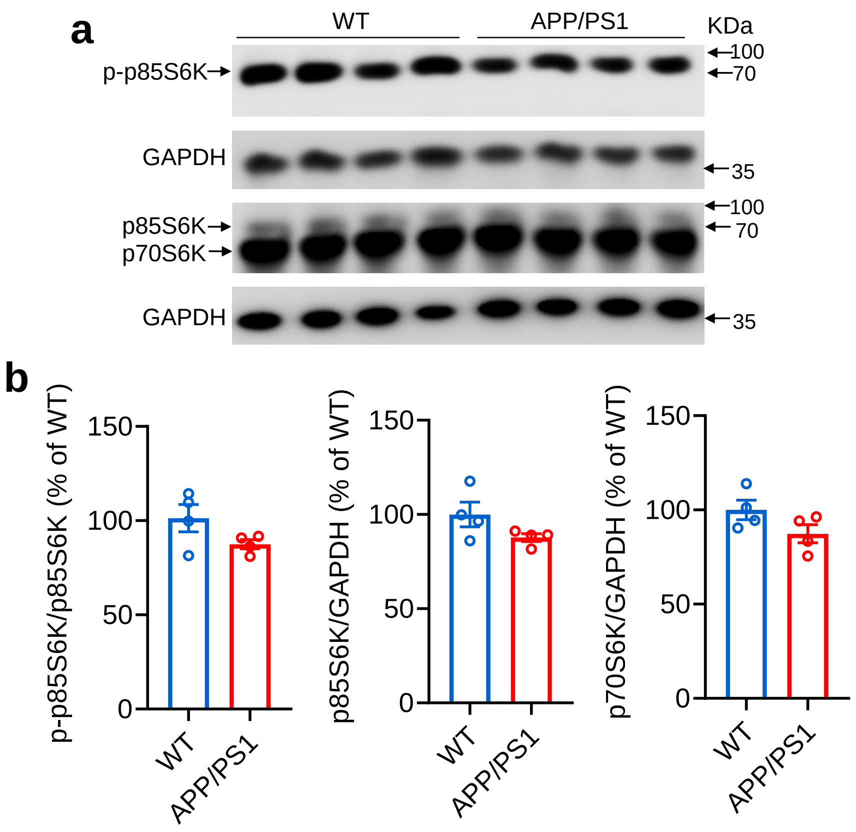

Fig. S7 p85S6K expression in P2 pellets of cortex of APP/PS1 mice. **a, b** The expression of phosphorylated p85S6K, p85S6K and p70S6K in P2 from fractionation of cortex of 9-month old APP/PS1 mice. n = 4 mice per group. t = 1.800, df = 6, P = 0.1219 for p-p85S6K, t =1.772, df = 6, P = 0.1268 for p85S6K, t =1.791, df = 6, P = 0.1234 for p70S6K. Data are presented as mean ± SEM. Unpaired t test, two-tailed (**b**). $^{*}P < 0.05$.

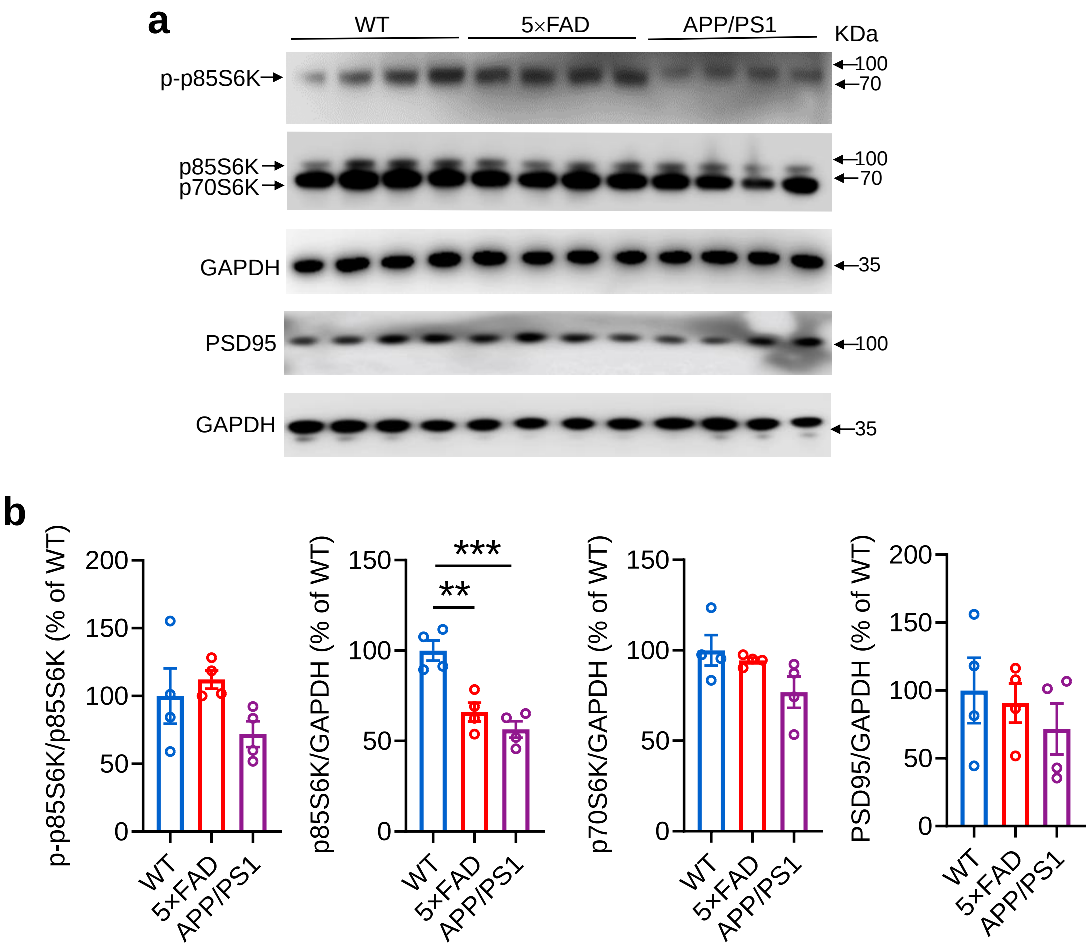

Fig. S8 p85S6K expression is decreased in PSD-1 of cortex of 5×FAD mice. **a, b** The expression of p85S6K/p70S6K, phosphorylated p85S6K and PSD95 in PSD-1 from fractionation of cortex of 7-month old 5×FAD mice. n = 4 mice per group. $F = 2.322$, $P = 0.158$ for p-p85S6K, $F = 19.75$, $P = 0.0005$ for p85S6K, $F = 2.937$, $P = 0.1043$ for p70S6K and $F = 0.5528$, $P = 0.5937$ for PSD95. Data are presented as mean ± SEM. Ordinary one-way ANOVA followed by Tukey's test. $^{**}P < 0.01$, $^{***}P < 0.001$.

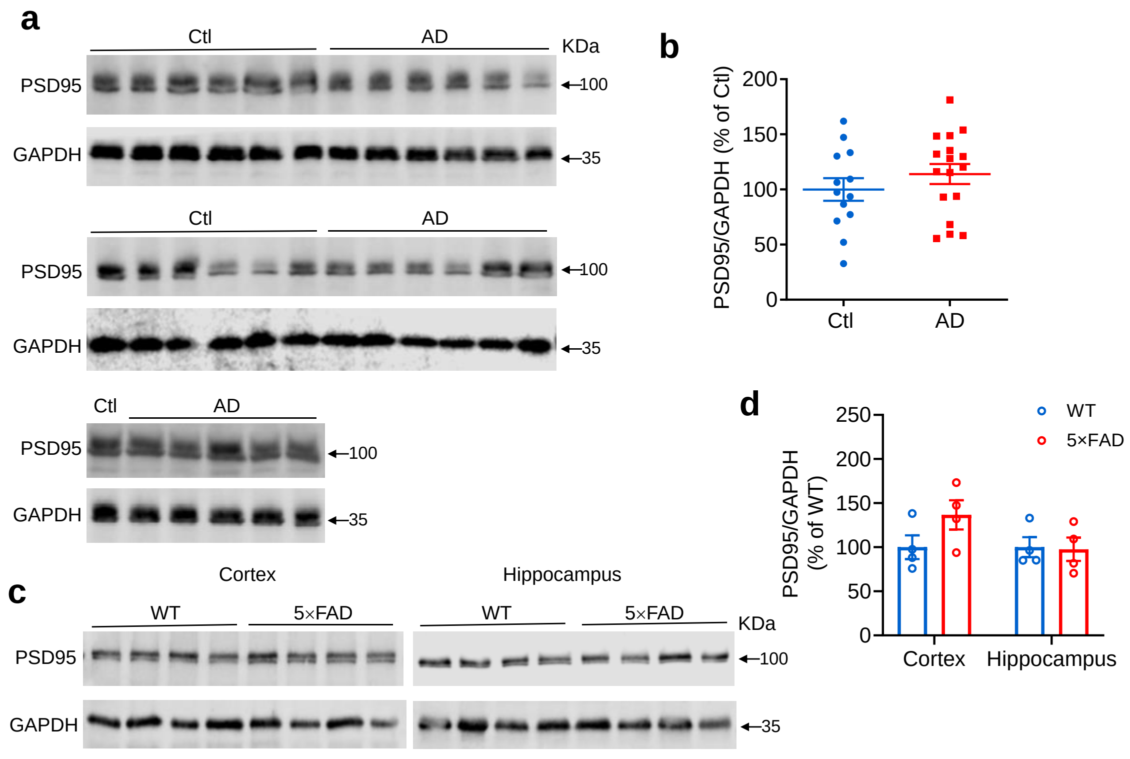

Fig. S9 PSD95 expression is not altered in AD brains. **a, b** The expression of PSD95 in P2 pellets from fractionation of postmortem temporal cortex from human AD brains and non-demented control (Ctl). n = 13 for Ctl and n = 17 for AD. t = 1.020, df = 28, *P* = 0.3164. **c, d** The expression of PSD95 in P2 pellets from fractionation of cortex and hippocampus of 7-month old 5×FAD mice. n = 4 mice per group. *t* = 1.681, df = 6, *P* = 0.1438 for cortex, *t* = 0.1346, df = 28, *P* = 0.8974 for hippocampus. Data are presented as mean ± SEM. Unpaired *t* test, two-tailed.

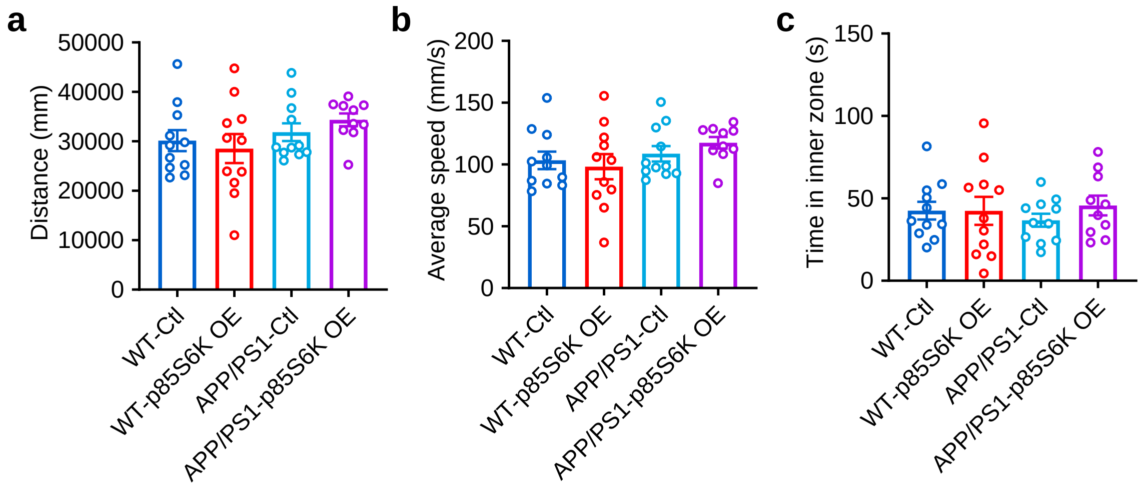

Fig. S10. Overexpression of p85S6K does not affect the locomotor activity and anxiety-like behavior. **a** Distance moved in open field test. $F_{(1, 39)}$ =3.083, P = 0.0870 for genotype, $F_{(1, 39)}$ = 0.04401, P = 0.8349 for p85S6K expression manipulation. **b** Average speed of mice in open field test. **c** Time spent in the inner zone in open field test. $F_{(1, 39)}$ = 0.04231, P = 0.8381 for genotype, $F_{(1, 39)}$ = 0.4950, P = 0.4859 for p85S6K expression manipulation. n = 11 mice for WT-Ctl, WT-p85S6K OE, APP/PS1-Ctl and n = 10 for APP/PS1-p85S6K OE. Data are presented as mean ± SEM. Two-way ANOVA.

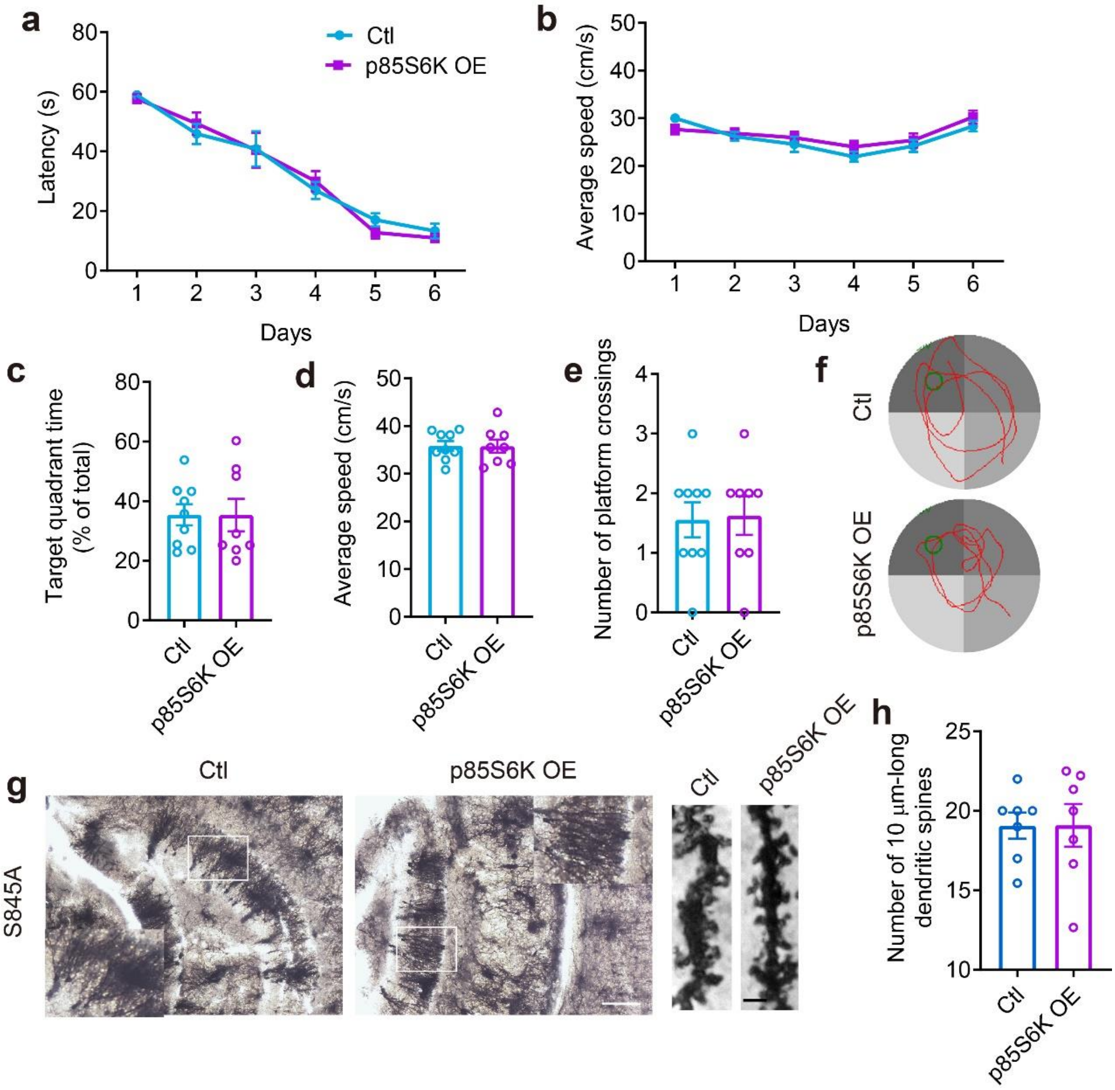

Fig. S11 Upregulation of p85S6K does not enhance the spatial learning and spine density in S845A mice. **(a-f)** MWM was performed to examine the effect of p85S6K upregulation on spatial learning in S845A mice. **a** Latency of S845A mice to locate the hidden platform in the training period in MWM test. $F_{(1, 15)} = 0.009521$, $P = 0.9236$. **b** The average speed of S845A mice in the training period. **c** The time of S845A mice spending in the target quadrant in the probe test. $t = 0.01373$, df = 15, $P = 0.9892$. **d** The average speed of S845A mice in the probe test. **e** The number of platform crossings of S845A mice in the probe test. $t = 0.1591$, df = 15, $P = 0.8757$. **f** Representative swimming trajectories in the probe test from different group of mice. The green circle

represented place for the hidden platform. n = 9 mice for Ctl and n = 8 for p85S6K OE (a-f). **(g, h)** Spine density in hippocampus of S845A mice after overexpression of p85S6K. Scale bar: 200 μm and 1 μm. n = 7 slices from 3 mice per group. t = 0.01436, df = 12. P = 0.9888. Data are presented as mean ± SEM. Repeated measure two-way ANOVA (**a, b**) and unpaired t test, two-tailed (**c, d, e, h**).

query :
MRRRRRRDGFYLAPDFRHREAEDMAGVFDIDLDQPEDAGSEDELEEGGQLNESMDHGGVG
PYELGMEHCEKFEISETSVNRGPEKIRPECFELLRVLGKGGYGKVFQVRKVTGANTGKIF
AMKVLKKAMIVRNAKDTAHTKAERNILEEVKHPFIVDLIYAFQTGGKLYLILEYLSGGEL
FMQLEREGIFMEDTACFYLAEISMALGHLHQKGIIYRDLKPENIMLNHQGHVKLTDFGLC
KESIHDGTVTHTFCGTIEYMAPEILMRSGHNRAVDWWSLGALMYDMLTGAPPFTGENRKK
TIDKILKCKLNLPPYLTQEARDLLKKLLKRNAASRLGAGPGDAGEVQAHPFFRHINWEEL
LARKVEPPFKPLLQSEEDVSQFDSKFTRQTPVDSPDDSTLSESANQVFLGFTYVAPSVLE
SVKEKFSFEPKIRSPRRFIGSPRTPVSPVKFSPGDFWGRGASASTANPQTPVEYPMETSG
IEQMDVTVSGEASAPLPIRQPNSGPYKKQAFPMISKRPEHLRMNL

Final results predicted by INSP

Predicted NLS peptide	START	END	SCORE
MRRRRRRDGFYLAPDFRHREAEDM	1	24	0.9419
PKIRSPRRF	430	438	0.93776

The model predicts NLS by the consensus model combined by large-scale frequent pattern mining model and statistical knowledge-based and machine learning SVM-based model with merging (threshold: 0.929).

Statistical knowledge-based and machine learning SVM-based model without merging

Predicted NLS peptide	START	END	SCORE
RRRRRRDGFY	2	11	0.9419
MRRRRRRDGFY	1	11	0.94009
MRRRRRRDGFYL	1	12	0.9379
PKIRSPRRF	430	438	0.93776
MRRRRRRDGFYLAPDFRHR	1	19	0.93681

The model selects top5 NLSs predicted by our statistical knowledge-based and machine learning SVM-based model without merging the fragments.

Large-scale frequent pattern mining model

Predicted NLS peptide	START	END	ENRICH	SCORE
MRRRRRR	1	7	0.791	0.78904
RRRRRR	2	7	0.923	0.78056
RRRRR	2	6	0.608	0.77464
RSPRR	433	437	0.646	0.62686

The model selects top3 NLSs in the enrichment score and top3 in the ranking score from the potential NLS database mined by large-scale frequent pattern mining model.

Fig. S12 The NLS predicted in p85S6K by www.csbio.sjtu.edu.cn/bioinf/INSP/.

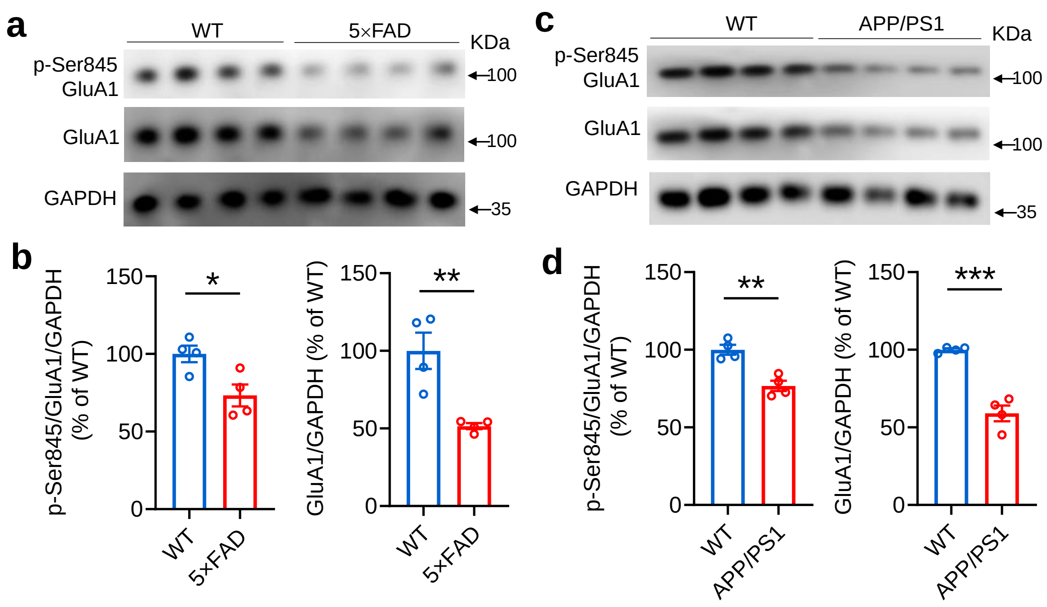

Fig. S13 The expression of GluA1 and its phosphorylation at Ser845 are reduced in P2 pellets from fractionation of cortex of 7-month old 5×FAD (**a, b**) and 9-month old APP/PS1 (**c, d**) mice. n = 4 mice per group. 5×FAD: $t = 3.023$, df = 6, $P = 0.0233$ for p-Ser845, $t = 4.104$, df = 6, $P = 0.0063$ for GluA1; APP/PS1: $t = 5.133$, df = 6, $P = 0.0022$ for p-Ser845, $t = 7.981$, df = 6, $P = 0.0002$ for GluA1. Data are presented as mean ± SEM. Unpaired t test, two-tailed (**b**, **d**). $^{*}P < 0.05$, $^{**}P < 0.01$, $^{***}P < 0.001$.
